# Site-Specific Cassette Exchange Systems in the *Aedes aegypti* Mosquito and the *Plutella xylostella* Moth

**DOI:** 10.1371/journal.pone.0121097

**Published:** 2015-04-01

**Authors:** Roya Elaine Haghighat-Khah, Sarah Scaife, Sara Martins, Oliver St John, Kelly Jean Matzen, Neil Morrison, Luke Alphey

**Affiliations:** 1 Department of Zoology, University of Oxford, Oxford, United Kingdom; 2 Oxitec Limited, Oxford, United Kingdom; New Mexico State University, UNITED STATES

## Abstract

Genetically engineered insects are being evaluated as potential tools to decrease the economic and public health burden of mosquitoes and agricultural pest insects. Here we describe a new tool for the reliable and targeted genome manipulation of pest insects for research and field release using recombinase mediated cassette exchange (RMCE) mechanisms. We successfully demonstrated the established ΦC31-RMCE method in the yellow fever mosquito, *Aedes aegypti*, which is the first report of RMCE in mosquitoes. A new variant of this RMCE system, called iRMCE, combines the ΦC31-*att* integration system and Cre or FLP-mediated excision to remove extraneous sequences introduced as part of the site-specific integration process. Complete iRMCE was achieved in two important insect pests, *Aedes aegypti* and the diamondback moth, *Plutella xylostella*, demonstrating the transferability of the system across a wide phylogenetic range of insect pests.

## Introduction

The ability to introduce exogenous DNA segments into predetermined locations in insect genomes is crucial for insect synthetic biology. In particular, it is important for insects intended for potential field release that the synthesis method can exclude extraneous sequences such as antibiotic resistance genes and other plasmid-derived sequences not required in the final product[1].

Currently, non-autonomous transposable elements, such as *piggyBac*, are widely used for germ-line transformation of insects. The *piggyBac* transformation system inserts transgenes specifically into TTAA tetranucleotide target sites[2]. These are present in large numbers in insect genomes, leading to partially-random integration of *piggyBac*-based constructs. Insertion at different sites may have different fitness costs[3], furthermore the phenotypic expression of a transgene is affected by its insertion site, a phenomenon known as a genomic position effect[2]. Consequently, partially-random insertion allows the production of a panel of lines with independent insertion events having slightly different properties, from which lines with the preferred properties can then be selected. In contrast, re-use of the same insertion site would allow better comparability and consistency between insertions of different constructs, and a deep knowledge base could be developed for selected insertion sites[4]. This would be highly desirable for synthetic biology, with its emphasis on standardisation and characterisation of genetic elements.

The ΦC31-*att* system involves recombination between the specific attachment sites (*attP* and *attB*), producing hybrid sites *attL* and *attR*[5]. These hybrid sites are no longer recognised by the integrase making the integration stable and efficient for transgenesis. The specific target sequences are sufficiently complex that they do not occur naturally in insect genomes, though ‘pseudo-*attP* sites’ exist in *Ae*. *aegypti* that allow off-target insertions at very low frequency[6]. This site-specific integration systems require the initial integration of the target sequence into the host’s genome as a ‘docking site’. Transgenes of interest can then be inserted into the ‘docking site’ in the presence of the appropriate recombinase. Differences attributed to the transgenic modifications can be directly compared since position effects would remain constant between integrated transgenes.

ΦC31-*att* integration has been successfully demonstrated in a diverse range of species, including fruit flies[4,7], mosquitoes[6,8,9], moths[10], human cells[11], plants[12], mice[13] and zebrafish[14]. Despite its transferability, a limitation of the ΦC31-*att* system is that the whole *attB* plasmid inserts into the insect genome including antibiotic resistance genes residing in the plasmid backbone. For insects intended for potential field release in particular, it is important that the integration method excludes sequences such as antibiotic resistance genes and other plasmid-derived sequences not required in the final product[15–18].

To overcome this, recombinase-mediated cassette exchange (RCME) is of great value[19,20]. RMCE is an integration strategy that makes use of site specific recombinases, in which a donor sequence and a target sequence are both flanked by site-specific recombination sites. Double recipricol recombination between the two sites results in the integration of the DNA segment between them as long both sites are heterospecific (cannot recombine together).

RMCE can be achieved by using inverted pairs of recombination sites flanking a cassette to be inserted. This cassette may be interchanged with another, flanked by the corresponding recomination sites that has been pre-inserted into the genome, in the presence of the recominase[19]. Since only the cassette of interest is inserted, no extraneous sequence need be included in the final insertion. This sequence conservative integration makes RMCE an attractive strategy for site-specific genome engineering of insects intended for field release.

The recombinases widely used for RMCE are the Cre[21] and FLP[22] recombinases, which promote recombination between the short nucleotide sequences *loxP* (34 bp) and *FRT* (48 bp) respectively. The recombination reaction is reversible in the presence of these recombinases, such that the rate of RMCE is low, whilst recombination is hampered by the thermodynamically disfavoured insertion of any donor cassette larger than the acceptor cassette[19]. As such, the use of Cre and FLP recominases is undesirable for the creation of efficient and stable RMCE systems. RMCE mediated by Cre-*loxP*[21] or Flp-*FRT*[22] systems have been demonstrated in mammalian cells[23–25], plants[26] and prokaryotes[27,28]. In insects, Cre- and Flp-RMCE was developed in *Drosophila melanogaster*[29,30]. Flp-RMCE was also shown in the silkworm, *Bombyx mori* [31]and Cre-RMCE in the tephritid fuit fly, *Anastrepha suspense* [32].

ΦC31 site-specific integrase can overcome the problem of poor integration efficiency encountered by reversble recombinases because the *attL* and *attR* sites formed after recomination are inert to further recombinase exposure. An efficient RMCE system using ΦC31-integrase (ΦC31-RMCE) was developed to allow for negative marker selection of transgenic *D*. *melanogaster* using two pairs of inverted recombination sites [33]. ΦC31-RMCE was also shown in the silkworm, *Bombyx mori*[34].

We tested the transferability of the ΦC31-RMCE system to mosquitoes by developing the system in the dengue-transmitting mosquito, *Ae*. *aegypti*. Furthermore, we developed an alternative two-step integration-excision RMCE system, integrase-recombinase mediated cassette exchange (iRMCE), which utilises ΦC31-*att* integration[5] of exogenous genetic material, followed by Cre*-loxP* or FLP-*FRT* excision of plasmid backbone sequences. Components of this novel system are interchangeable, and experiments were conducted to demonstrate the reproducibility of this new iRMCE system in two different insect species, the *Ae*. *aegypti* mosquito, and the agriculturally important pest moth, *Plutella xylostella*.

## Materials and Methods

### Plasmid constructs

All plasmid sequences are provided in Table A in S1 File. Plasmids were constructed using standard molecular techniques from existing DNA constructs.

The OX4372 docking construct (*piggyBac*[*attP*-3xP3-DsRed2- *attP*]) was made by modifying the *pBac*[3xP3-ECFPaf]-*attP* plasmid[6] to contain a 3xP3-DsRed2, and to include the second inverted *attP*[11] site. The OX4476 docking construct *piggyBac*[*FRT*-*loxP*-3xP3-AmCyan-*attP*] was also made by modifying *pBac*[3xP3-ECFPaf]-*attP* plasmid[6] to contain the AmCyan reporter (BD Biosciences Clontech), and to include one *FRT*[35] and one *loxP*[29] site. The OX4540 docking construct *piggyBac*[*FRT*-*loxP*-OPIE2-DsRed-*attP*] was made by modifying the OX4476 plasmid to contain the OPIE2[36]-DsRed2 reporter instead of the 3xP3-AmCyan.

The donor constructs were made by modifying the *attB*[3xP3-DsRed2nls-SV40]lox66 plasmid[6] to include the *FRT*[35] and *loxP*[29] sites, and to change the reporters (3xP3-DsRed2 for OX4714, making *attB*[3xP3-DsRed2-SV40-*FRT*-*loxP*], and Hr5ie1-Zsgreen for OX4580, making *attB*[Hr5ie1-ZsGreen-SV40- *FRT*-*loxP*]). The donor OX4312 construct (*attB*[Hr5ie1-AmCyan]*attB*) was synthesised by modifying the *attB*[3xP3-DsRed2nls-SV40]lox66 plasmid[6], to include a second inverted *attB*[11] site and Hr5ie1[37]-AmCyan marker by conventional cloning methods.

### *Aedes aegypti* strains and rearing

The *Ae*. *aegypti* wild-type strain used was colonised in 1975 from Jinjang, Selangor, Malaysia. The strain was reared at 26°C (±1°C) and 70% (±10%) relative humidity with a 12:12 hour light:dark cycle. Larvae were fed on finely ground TetraMin Ornamental Fish Flakes (Tetra GmbH), and adults on a 10% sucrose solution with 14 U ml^-1^ penicillin and 14 μg ml^-1^ streptomycin. Adult females were fed on defibrinated horse blood screened for pathogens (TCS Biosciences).

### Plutella xylostella strains and rearing

The *P*. *xylostella* wild-type strain was obtained from Syngenta laboratories. The strain was reared at 27°C (±1°C) and 60% (±2%) relative humidity with a 12:12 hour light:dark cycle. Adults were fed 7.5% sucrose solution with 100 μg ml^-1^ tetracycline and 0.07% methylhydroxybenzoate. Eggs were collected on cabbage extract-coated Parafilm strips, which were moved to a plastic beaker containing beet armyworm artificial diet (catalogue number F9219B, Bio-serv).

### Generating transgenic strains

Injection solutions for transformation contained the transgenic DNA plasmid and the capped mRNA helper suspended in a buffer. The buffer had a final concentration of 5 mM KCl and 0.1 mM NaH_2_P0_4_, pH 6.8. DNA plasmids were purified using EndoFree Plasmid Maxi Kit (Qiagen). Helper *piggyBac* transposase transcribed from OX3081[8], site-specific ΦC31 integrase[6], the FLP[38] and Cre[39] recombinases were provided as capped mRNA transcribed with the mMESSAGE mMachine T7 Kit (Ambion) and purified using the MEGAclear Kit (Ambion); sequences included the *Drosophila melanogaster vasa* gene’s 3’UTR[40].

Injection solutions for *P*. *xylostella* contained: 500 ng μl^-1^ transgenic DNA plasmid with 300 ng μl^-1^ capped mRNA helper for *piggyBac* transformation, and 350 ng μl^-1^ transgenic DNA plasmid with 500 ng μl^-1^ capped mRNA helper for ΦC31-*att* integration. Injection solutions for *Ae*. *aegypti* contained: 300 ng μl^-1^ transgenic DNA plasmid with 700 ng μl^-1^ capped mRNA helper for *piggyBac* transformation, and 300 ng μl^-1^ transgenic DNA plasmid with 730 ng μl^-1^ capped mRNA helper for ΦC31-*att* integration. 700 ng μl^-1^ capped mRNA helper was used for either Cre or FLP excision in both insects.

Pre-blastoderm *Ae*. *aegypti* embryos were microinjected as described[41] using a BA400 Motic light microscope (Microscope Services Ltd), an MN-151 micromanipulator (Narishige), and an Eppendorf FemtoJet microinjector air-pump (Eppendorf). To remove the oil, cover slips of the newly injected embryos were placed vertically in water for up to three hours, and then placed in fresh water for four days, giving the embryos sufficient time to fully develop before hatching.

Pre-blastoderm *P*. *xylostella* embryos were injected as described[42]. Glass slides of newly injected embryos were placed in a sealed box for ~48 hours, then transferred to a beaker containing 30 ml of the beet armyworm artificial diet to hatch.

Up to 100 female G0s were pooled in cages with 1:1 wild-type males and their offspring were screened for the transgenic marker. For ΦC31-mediated integration and ΦC31-RMCE, 2–3 G0 males were pooled in small pots with wild-type females (1:10 males:females). After three days, insects from up to ten pots were pooled into each cage and offspring from these cage were screened for the transgenic marker. For Cre or FLP injections, males were pooled into 1–4 G0 males per pot with wild-type females (1:10 males:females) and offspring were collected from these pots for screening. Female G0 survivors from the same injections were pooled together into cages with up to 100 G0 females as before with wild-type males (1:1). Following a blood meal, these G0 females were individually placed in tubes for egg collection, and all offspring were screened. In all cases, G1 offspring were screened from two gonotrophic cycles.

For ΦC31-mediated integration and ΦC31-RMCE, and for Cre/FLP-mediated excision, a mixure of hemizygous and homozygous embryos were injected with the recombinase and donor construct. G0s lacking any transgenic marker were discarded.

To identify transgenic insects, third instar G1 larvae or pupae—offspring of injected G0 embryos—were screened for the presence of fluorescent markers using a Leica MZFLIII or Olympus SZX12 fluorescence microscope with the appropriate filter sets—ECFP (blue) exciter D436/20x; emitter D480/40m; DsRed2 (red): exciter HQ545/30x; emitter HQ620/60m); GFP (green): exciter 475/15; emitter 510LP—from Chroma Technology. All images were taken with the Olympus SZX12 fluorescent microscope using a Canon PowerShot S5IS and an MM99 adaptor (Martin microscopes).

The number of recombination events were defined as the number of G0 pools that produced transgenic offspring. Therefore potential multiple transgenic G1s from the same G0 pool were conservatively assumed to represent only a single transformation event from one G0 parent.

We calculated the minimum recombination efficiencies as the proportion of injected survivors (G0s) that produced transgenic offspring. This results in a lower calculated efficiency than if only fertile G0 individuals were considered. Multiple transgenic G1s from the same G0 parent or pool were assumed to represent a single recombination event.

### Genomic DNA exctraction

Genomic DNA was isolated from single insects using the Machery-Nagel Nucleospin Kit (Machery-Nagel) according to the manufacturer’s protocol and eluted in 50 μl endonuclease free water.

### Adaptor-based amplification of flanking genomic nucleotide sequences

To obtain the genomic nucleotide sequences flanking the transposed *piggyBac*-based construct, an adaptor-mediated PCR amplification technique[42] was performed using primers specific for the adaptor and *piggyBac* sequence (Table B in S1 File). PCR products were cloned using the GeneJET PCR Cloning Kit (Fermentas), and resulting minipreps sequenced by GATC Biotech.

### PCR analysis of integration and excision events

PCRs were carried out using DreamTaq polymerase (Fermentas) according to the manufacturer’s protocol, with 35 cycles of: 15 seconds at 94°C; 30 seconds at 55–60°C (primer-dependant); 72°C for 30 seconds per DNA kilobase, and; 7 minutes at 72°C. Primers are listed in Table C in S1 File.

## Results

### Development of RMCE docking lines

Wild-type *Ae*. *aegypti* embryos were co-injected with a docking construct and capped *piggyBac* transposase mRNA (embryos injected: 1000, G0 survival: 25%. Three independent *Ae*. *aegypti* docking strains for ΦC31-RMCE (OX4372A, OX4372F, and OX4372I) were developed that showed 1:1 transgenic:non-transgenic Mendelian inheritance pattern of the docking constructs’ fluorescent markers consistent with the presence of a single insertion.

Six independent *Ae*. *aegypti* OX4476 docking strains for iRMCE (embryos injected: 2000, G0 survival: 25%) were developed, each originating from separate G0 pools. Of the six *Ae*. *aegypti* OX4476 strains, one had a fitness cost associated with the transgene (only 18% of emerging adult offspring of hemizygous parents were transgenic, rather than the expected 50%), and one had multiple insertions. The remaining four showed typical 1:1 Mendelian inheritance of the construct. *Ae*. *aegypti* OX4476C, OX4476F, and OX4476H strains were selected for use in subsequent experiments.

Wild-type *P*. *xylostella* embryos were co-injected with docking construct OX4540 and the *piggyBac* transposase helper plasmid[42] (embryos injected: 1868, G0 survival: 47%); post-injection survival was equivalent to that previously reported[42]. Out of 22 independent *P*. *xylostella* OX4540 strains obtained, two were sterile and Mendelian inheritance data showed that five had multiple insertions and two had a lower proportion of transgenic progeny than non-transgenic progeny (39% and 42% transgenic progeny), which may indicate a fitness cost associated with the transgene. The remaining thirteen strains showed a typical 1:1 Mendelian inheritance of the construct and were therefore thought to have only one insertion. *P*. *xylostella* OX4540A was picked randomly from these thirteen strains.

Genomic sequences flanking the transgenic docking constructs were determined for all transgenic strains except *Ae*. *aegypti* OX4372F, *Ae*. *aegypti* OX4476H, and *P*. *xylostella* OX4540A (Table 1), which are thought to have inserted into highly repetitive sequences that are difficult to amplify[43]. Flanking sequences were different in each strain and, as expected[44], all carried the TTAA tetranucleotide sequence at the construct-genomic junctions. Obtained sequences were compared to the published *Ae*. *aegypti* sequence databases, and all sequences aligned to genomic regions with no known coding sequences.

**Table 1:** Genomic flanking sequences of OX4372 and OX4476 in *Ae*. *aegypti*.

Line	5’-genomic flanking sequence	3’-genomic flanking sequence
ΦC31-RMCE		
OX4372A	TTTTGTGTAAGTTTTGTATTTTAA	TTAAGGAGTTCAACCTGCAAACCT
OX4372F	Not determined	Not determined
OX4372I	TCTATCAGTATACATTGGCCTTAA	TTAAATCTAAAGAACTGCTACGAT
iRMCE		
OX4476C	TTTCCATACAGACTTAACATTTAA	TTAAAAAAGGCCATGTTTTAGTGCT
OX4476F	GAGCGATGTTAAAGTTTGTTTTAA	TTAATTAGTGTCTGATTCTGGGAAT
OX4476H	Not determined	Not determined

The duplicated TTAA *piggyBac-*insertion site was present in all 5’ and 3’ insertion boundaries (underlined).

### ΦC31-RMCE in *Ae*. *aegypti*

*Ae*. *aegypti* OX4372 docking strains were co-injected with donor plasmid OX4312 and capped ΦC31 integrase mRNA. G0s were pooled and minimum recombination efficiency was calculated (Table 2). The number of recombination events were defined as the number of G0 pools that produced transgenic offspring carrying the Hr5ie1-AmCyan marker (blue bodies). Potential multiple RMCE G1s from the same G0 pool were conservatively assumed to represent only a single RMCE event from one G0 parent.

**Table 2:** Summary of *Ae*. *aegypti* OX4312 injections for ΦC31-RMCE.

Donor plasmids	Docking lines	Embryos injected	G0 adults	R	Efficiency
OX4312	OX4372A*	1594	170 [10.7%]	7	4.1%
	OX4372F	1450	242 [16.7%]	0	
	OX4372I	2158	462 [21.4%]	22	4.8%

*All G1 transformants from OX4312 injections into OX4372A resulted in incomplete cassette exchange due to only a single pair of attachment sites recombining. Embryos of this partially recombined line, called ‘OX4372A[OX4312]-incomplete’ were injected with additional ΦC31 recombinase mRNA resulting in a second recombination step in G1 progeny (Efficiency = 18.5%).

Recombination events (R) are defined as the number of transgenic pools. Efficiency = minimum calculated integration efficiency.

No ΦC31-mediated integration was indicated in G1 offspring from injected OX4372F insects.

**Fig 1:**
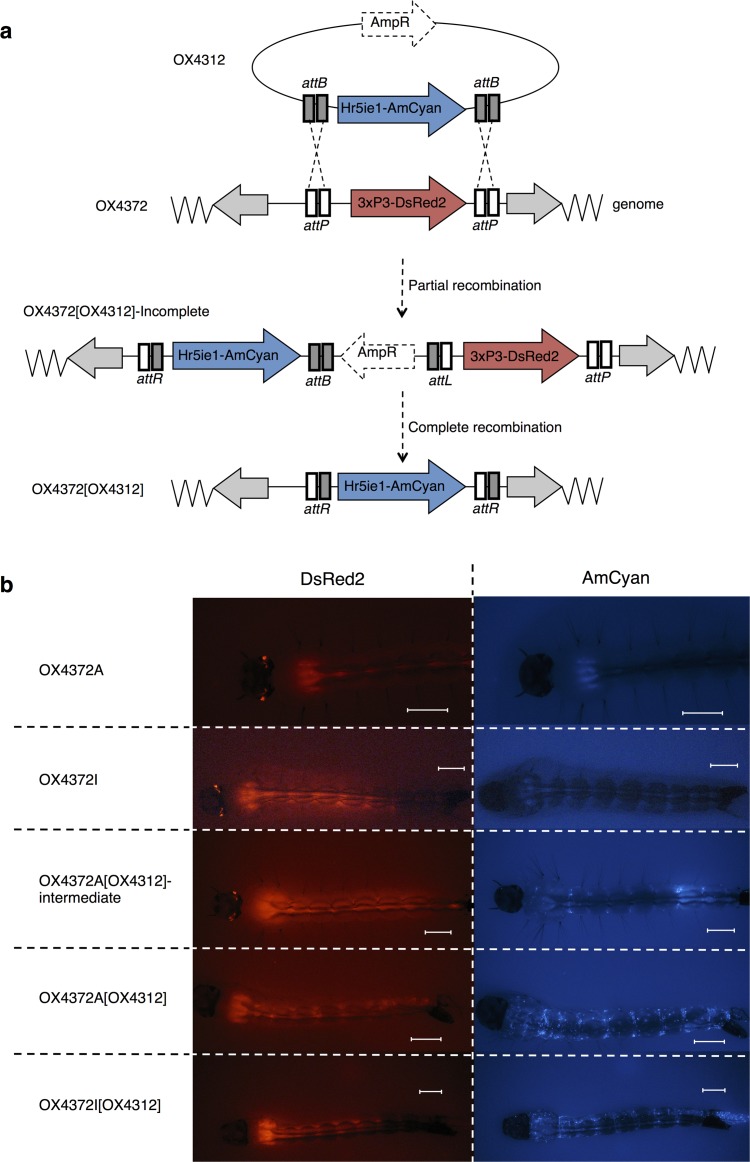
ΦC31-RMCE in *Ae*. *aegypti*. (a) Schematic diagram of recombination between constructs OX4372 and OX4312 in ΦC31-RMCE, and (b) fourth instar larval expression of fluorescent reported genes in *Aedes aegypti* OX4372 lines before and after ΦC31 mediated recombination with construct OX4312. OX4372A and OX4372I strains showed only 3xP3-DsRed2 (red eyes). Following recombination in the presence of the ΦC31-integrase, one set of attachment sites resulted in the expression of both 3xP3-DsRed2 (red eyes, shown with white arrows in top right panel) from the docking construct OX4372 and the Hr5ie1-AmCyan (blue bodies) from the donor construct OX4312 (OX4372A[OX4312]-incomplete). Recombination of both sets of ΦC31 attachment sites (*attB* and *attP*) resulted in complete RMCE, where only the AmCyan reporter (blue bodies) was observed in OX4372A[OX4312] and OX4372I[OX4312]; the 3xP3-DsRed2 marker for red eyes was excised in these insects (heads are to the left of images; diffuse red staining in larval thorax and abdomen is due to autofluorescence). Scale bars represent 1mm for each image.

The cuticular expression of the AmCyan and the absence of the DsRed2 marker in OX4372I G1 offspring (Fig 1) indicated germline recombination between both pairs of attachment sites and thus complete cassette exchange, creating new strain OX4372I[OX4312]. This was confirmed by amplifying and sequencing across both ΦC31 attachment sites and fluorescent marker junctions (Fig A in S1 File).

All G1 transformants from OX4312 injections into OX4372A resulted in the presence of cuticular expression of DsRed2 and patchy AmCyan expression in the optic nerves. This indicated incomplete cassette exchange due to recombination of only a single pair of attachment sites (Fig 1b), creating ‘OX4372A[OX4312]-incomplete’. This incomplete recombination event was confirmed by positive amplification of the intact *attP*-DsRed2 3’ junction, and also of the recombined *attR*-AmCyan 5’ junction (see Fig A in S1 File). The OX4372A[OX4312]-incomplete strain showed reduced expression of the docking cassette’s DsRed2 marker, as we have often seen in other ΦC31 integrations of complete plasmids leading to insertions carrying two 3xP3-based markers[8].

Individuals of this partially recombined line, OX4372A[OX4312]-incomplete, were crossed to each other and their offspring, a mixure of hemizygous and homozygous embryos, were injected with additional ΦC31 integrase mRNA. This resulted in loss of the DsRed2 marker and brighter expression of the AmCyan, which indicated a second recombination event between the remaining *attB* and *attP* sites, resulting in complete cassette exchange and creating OX4372A[OX4312].

Recombination efficiency was much higher during this second round of injection at 18.5%, perhaps due to the close proximity of the two attachment sites, such that the recombinase had a smaller search volume of potential sites. Complete cassette exchange was confirmed in line *Ae*. *aegypti* OX4372A[OX4312] by positive amplification followed by sequencing of the recombined *attR*-AmCyan 5’ and 3’ junctions, and absence of the *attP*-DsRed2 5’ and 3’ junctions (see Fig A in S1 File).

### iRMCE:ΦC31-integration followed by Cre/FLP excision

#### Phase I: ΦC31-mediated integration

Docking strains were co-injected with the donor construct and capped ΦC31 integrase mRNA for ΦC31-mediated integration.

*Aedes aegypti*. The G0 *Ae*. *aegypti* OX4476 docking strains injected with OX4714 were pooled and minimum recombination efficiency calculated (as previously described), Table 3.

**Table 3:** Summary of *Ae*. *aegypti* and *P*. *xylostella* injections for ΦC31-*att* integration.

Donor plasmids	Docking lines	Embryos injected	Surviving G0 adults	I	Efficiency
*Ae*. *aegypti*					
OX4714	OX4476H	2507	230 [9.2%]	2	0.9%
	OX4476C	2968	280 [9.4%]	2	0.7%
	OX4476F	2698	273 [10.1%]	4	1.5%
*P*. *xylostella*					
OX4580	OX4540A	1206	365 [30.3%]	1	0.3%

Integration events (I) are defined as the number of transgenic pools. Efficiency = minimum calculated integration efficiency.

**Fig 2:**
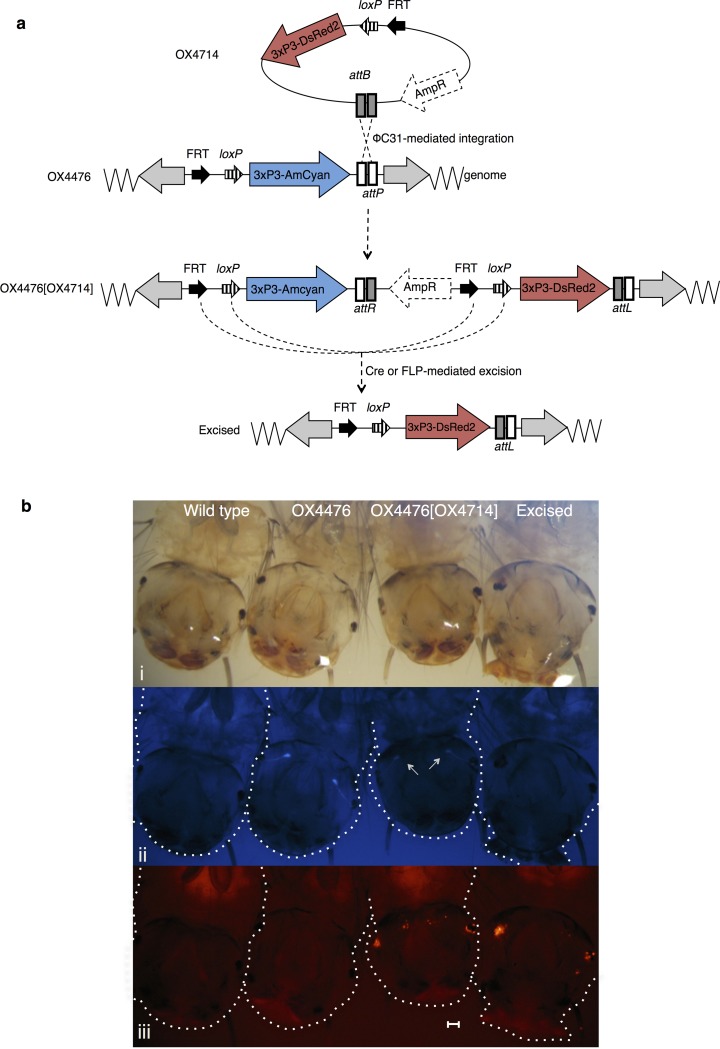
iRMCE in *Ae*. *aegypti*. (a) Schematic diagram of iRMCE in *Ae*. *aegypti* and (b) corresponding fluorescent phenotypes in fourth instar *Ae*. *aegypti* larvae following OX4714 injections. Black arrows indicate engineered *FRT* and *loxP* sites present in donor and acceptor constructs; AmpR represents the plasmid backbone sequences, which includes the ampicillin-resistance gene; grey arrows indicate *piggyBac* ends. The donor plasmid’s 3xP3-DsRed2 cassette is exchanged for the docking construct’s 3xP3-Amcyan. Larvae are shown under (i) white light, (ii) cyan, and (iii) red excitation light and filters. Images show complete exchange of the 3xP3-AmCyan cassette (OX4476), by integration of 3xP3-DsRed2 (seen in OX4476[OX4714]), and excision of the 3xP3-AmCyan marker (‘excised’ larva). There was reduced expression of the docking cassette’s 3xP3-AmCyan marker following ΦC31-*att* integration (white arrows: panel (ii), OX4476[OX4714]). Images (i-iii) were taken under the same magnification. Scale bar represents 0.5 mm. White dashed lines outline larvae.

Canonical ΦC31 integration of OX4714 into *Ae*. *aegypti* OX4476C and OX4476F lines (Fig 2) was observed by the presence of the eye-specific red and blue fluoresnent markers and was confirmed by positive PCR amplification, and subsequent sequencing of *attL* and *attR* junctions (Fig B in S1 File). ΦC31 integration of OX4714 into OX4476H was also confirmed (data not shown), however the integrated line produced very few offspring and was later lost.

ΦC31-mediated insertions do not absolutely require an *att*P site; even if one is present in the genome a proportion of insertions are found at other sites, so-called pseudo-*attP* sites[45]. Recombination of the donor construct’s *attB* site with endogenous sites that have little sequence identity with *attP* has been observed in *Ae*. *aegypti*, *D*. *melanogaster*, and mammalian cells[4,6,45,46]. Such non-*attP* insertions of OX4714 were observed in all injected *Ae*. *aegypti* OX4476 lines (H, C and F), indicated by the absence of the *attL* and *attR* junctions that are characteristic of ΦC31 integration (data not shown). These pseudo—*attP* insertions were discarded and are not included in the integration efficiency calculations.

**Fig 3:**
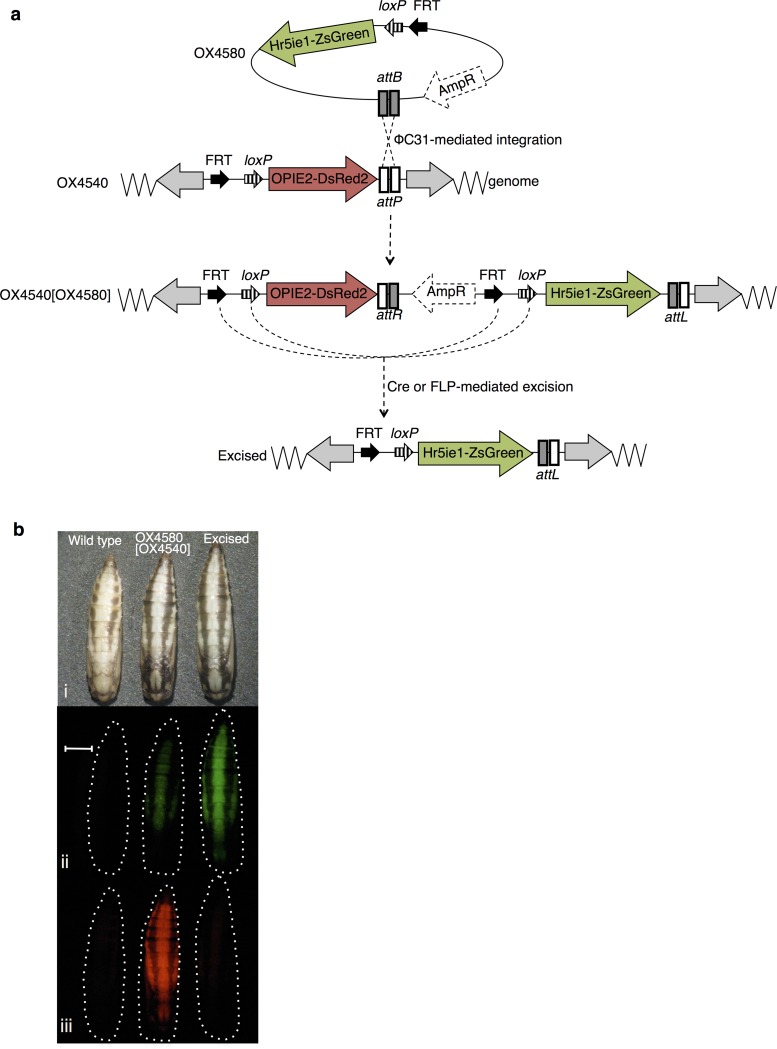
iRMCE in *P*. *xylostella*. (a) Schematic diagram of iRMCE in *P*. *xylostella* and (b) corresponding fluorescent phenotypes in *P*. *xylostella* pupae. Black arrows indicate engineered *FRT* and *loxP* sites present in donor and acceptor constructs; AmpR represents the plasmid backbone sequences, which includes the ampicillin-resistance gene; grey arrows indicate *piggyBac* ends. Pupae are shown under (i) white light, (ii) green excitation light and filters, and (iii) red excitation light and filters. Images (i–iii) were taken under the same magnification. Scale bar repersents 1 mm.

*Plutella xylostella*. A total of 1082 embryos from the docking strain OX4540A were injected with OX4580 and ΦC31-integrase mRNA; of these, 332 individuals survived to pupation. Crosses to wild-type were set up at the pupal stage. Their progeny were screened for ZsGreen expression (carried by OX4580). Out of 91 crosses, one generated individuals expressing ZsGreen. These were used to establish line OX4580[4540A] (Fig 3). The minimum calculated integration efficiency was 0.30%. Similar integration efficiencies (0.16% and 0.39%) for ΦC31 integration in *P*. *xylostella* were obtained previously in our lab (data not shown).

Canonical ΦC31 integration of OX4580 into *P*. *xylostella* OX4540A was confirmed by positive amplification and subsequent sequencing of *attL* and *attR* recombination junctions (see Fig C in S1 File).

#### Phase II: FLP- and Cre-mediated excision

As a result of the ΦC31 integration, the donor plasmid backbone and the marker cassette from the ‘docking’ construct in the *Ae*. *aegypti* OX4476[OX4714] strains F and C, and the *P*. *xylostella* OX4540A[OX4580] strain, are flanked by *loxP* and *FRT* sites in the same orientation. In the presence of Cre or FLP, excision of the flanked sequences, and thus loss of the docking-construct’s marker, was expected.

*Aedes aegypti*. To test excision efficiencies in *Ae*. *aegypti*, OX4476C[OX4714] and OX4476F[OX4714] lines were injected with FLP or Cre. Transgenic survivors were crossed to wild-type in small pools (2–5 injected G0s per pool). Individuals bearing excision events were identified through loss of the AmCyan marker (Fig 2). As anticipated, the number of false-positive excision events identified in the *Ae*. *aegypti* strains by fluorescence was high due to difficulty in correctly identifying AmCyan-positive (blue-eyed) individuals. PCR amplification across the excision junction followed by sequencing confirmed these excision events (see Fig B in S1 File). Excision did not appear to affect DsRed2 expression. For line OX4476F[OX4714], minimum excision efficiencies were estimated at 2.4% using Cre and 1.3% using FLP, whereas no excision was detected in line OX4476C[OX4714] (Table 4).

**Table 4:** Summary of *Ae*. *aegypti* and *P*. *xylostella* injections for Cre and FLP injections.

	Line injected	Embryos injected	Survived G0 adults	G0 pools	Marker loss	E	Efficiency
Cre	*Aae*.OX4476F[OX4714]	2520	205 [8.1%]	164	23	4	2.4%
	*Aae*.OX4476C[OX4714]	2570	312 [12.1%]	307	18	0	
	*Pxy*.OX4540A[OX4580]	1206	273 [22.6%]	83	75	75	90%
FLP	*Aae*.OX4476F[OX4714]	2650	247 [9.3%]	241	42	3	1.3%
	*Aae*.OX4476C[OX4714]	2535	263 [10.4%]	251	18	0	
	*Pxy*.OX4540A[OX4580]	1395	201 [14.4%]	63	63	63	100%

*Ae*. *aegypti* (‘*Aae*’) strains were injected with Cre and FLP in parallel for direct comparison. The number of *Ae*. *aegypti* pools with progeny apparently lacking the AmCyan marker (due to weak fluorescence) is shown (‘marker loss’); this was not a problem with the *P*. *xylostella* (‘*Pxy*’) strain so values in this column reflect true marker loss. Excision events (E) were confirmed by PCR and sequencing, and the minimum calculated excision frequencies (Efficiency) are shown as a percentage.

*P*. *xylostella*. Individuals bearing excision events were identified through loss of the DsRed2 marker (Fig 3). PCR amplification across the excision junction (Fig C in S1 File) followed by sequencing confirmed these excision events. The minimum Cre excision frequency was higher in *P*. *xylostella* OX4540A[OX4580] than in any of the *Ae*. *aegypti* lines, with 90% (75/83) of the G0 survivor pools producing at least one larva showing excision using the same Cre recombinase mRNA source as used in *Ae*. *aegypti* (Table 4). The excision frequencies were calculated per G0 family as the proportion of G1 offspring in which excision had occurred. In the 75 G0 families, excision occurred at frequencies between 0.88–100%.

Similarly, the same batch of FLP recombinase mRNA was used to inject *P*. *xylostella* and *Ae*. *aegypti*, albeit at different times and by different people. As with Cre, a higher minimum FLP-excision frequency was observed in *P*. *xylostella*. All 63 of the Go survivor pools produced at least one larva showing excision (Table 4). In these 63 families, excision occurred at frequencies of between 0.47%–100%.

In conclusion, full iRMCE was achieved in the two insect species; successful ΦC31 integration was followed by Cre excision and also by FLP excision in *Ae*. *aegypti* and in *P*. *xylostella*.

## Discussion

We successfully demonstrated two cassette exchange systems (ΦC31-RMCE and iRMCE) for the reliable and targeted transformation of insect species. We chose the *Aedes aegypti* mosquito and the diamondback moth (*Plutella xylostella*) due to their importance to agriculture and public health. This paper presents the first reported examples of RMCE in mosquitoes and in the pest moth *P*. *xylostella*. Furthermore, this is the first time that all three recombinases have been shown to work in *P*. *xylostella*, and that FLP has been shown to work in *Ae*. *aegypti*. Previous attempts at inducing FLP-mediated excision in *Ae*. *aegypti* were unsuccessful and included the use of a helper plasmid containing FLP under the control of the *D*. *melanogaster* hsp70 gene’s promoter[47]. It is unclear whether the T7 3’UTR or the use of FLP mRNA in this study aided the successful FLP-mediated excision.

We further showed that iRMCE docking lines *Ae*. *aegypti* OX4476C and OX4476F, and *P*. *xylostella* OX4540A, as well as ΦC31-RMCE docking lines *Ae*. *aegypti* OX4372A and OX4372I, all carry functional and accessible *attP*-docking sites that can be used to integrate a panel of genes into the insect’s genome. Further refinements to RMCE systems could also include incorporating methods to stabilise the *piggyBac*-based docking construct[48–52] to circumvent remobilisation risks for insect species in which remobilisation is known to occur, such as *Anopheles stephensi*[53].

The use of a single round of embryo injections makes the ΦC31-RMCE system less laborious for engineering transgenic insects. However, integration of transgenes in the first round, and subsequent excision of extraneous sequences in a separate step, as in iRMCE, is an attractive tool. Carefully designed donor cassettes could be used to compare transgenes in the presence or absence of additional elements that can later be excised in a separate step from the specific recombination sites. For example, a lethal transgene can be stably inserted into an insect genome if it contains its genetic repressor in the construct. Subsequently, this genetic repressor can be excised if flanked by two *FRT* or *loxP* sites.

iRMCE worked well in two Orders of insects, indicating the robustness of the system. However, several elements may benefit from optimisation. For example, improved integration efficiency of ΦC31 integrase with the addition of a nuclear localised signal to the C-terminus (NLS-C) was shown in mammalian cells[54] and *D*. *melanogaster*[55], though not in *P*. *xylostella* (data not shown). Alternatively, the use of ΦC31 ‘self-docking’ strains drastically improved the efficiency of the ΦC31-*att* system in *Anopheles gambiae*[55,56], and the use of *hsp70* gene promoter and heat-shocking embryos can induce high Cre-mediated excision frequencies in *D*. *melanogaster*[57] and *Ae*. *aegypti*[47], but failed to induce FLP-mediated excision in *Ae*. *aegypti*[47].

In *Ae*. *aegypti*, ΦC31-*att* integration resulted in reduced expression of the docking cassette’s fluorescent markers, consistent with previous observations in *Aedes albopictus*[8]. This effect may be due to transcriptional interference[58,59] or changes in chromatin structure resulting in polymerase interference[60,61], and indicates that even a modest change at the same site can affect transgene expression levels.

The variation in ΦC31 integration and FLP/Cre-mediated excision frequencies between docking lines carrying the same engineered molecular constructs suggests that the structure of flanking genomic sequences also affects the efficiency of these systems. Previous work in *D*. *melanogaster*[55], *Ae*. *aegypti*[6] and *Ae*. *albopictus*[8] also suggests that the efficiency of ΦC31-*att* integration is dependent on the chromosomal location of the attachment sites, though the precise molecular basis is not yet clear.

The ability to directly compare and characterise components (such as lethal, suppressor or refractory effector elements) facilitates the fine-tuning of expressed phenotypes. This minimises the time-consuming nature of transforming and characterising transgenic insects using *piggyBac*-based transposons as otherwise a panel of insertion lines needs to be generated and analysed for each construct to understand the ‘average’ behaviour. However, without an effective cassette exchange system, organisms engineered using site-specific serine-integrases such as ΦC31-*att* will carry plasmid backbone sequences, including antibiotic resistance genes, introduced as part of the integration mechanism. This is undesirable, especially for strains potentially intended for field release.

Use of meganucleases such as TALENs and CRISPR/Cas9[62] may allow targeted integration of a molecular construct at any genomic locus without inserting the extraneous plasmid sequences. Since integration efficiency may be low, insertion of an *attP* site may be attractive, and facilitate re-use of the insertion site. Targeted integration using a meganuclease was recently demonstrated in *An*. *gambiae*[63], albeit using a transgenic rather than endogenous target site. Though such sites, or pre-existing *attP* insertions, may not have the adjacent *loxP* or FRT sites, unwanted parts of the *attB* plasmid may still be removed post-integration if flanked by paired *loxP* or FRT sites.

A key tenet of synthetic biology is the use of standardised, characterised, modular components. One overlooked element of this is the insertion site[52]. The ability to re-use insertion sites will encourage the development of a set of insertions sites of known properties, with each further use of this set contributing further to the dataset. Desirable properties for an insertion site vary somewhat between applications, but baseline characterisation may include assessment of fitness, effects on life history parameters such as size, development time, longevity, male mating competiveness and female fecundity and fertility, as well as molecular characterisation. Pre-selection of insertion sites with appropriate characteristics in this regard would significantly facilitate the development of functional strains, including for potential field use.

## Supporting Information

S1 File File containing Figures A–C, and Tables A–C. (PDF)
