# Supplementary material for: Site-Specific Cassette Exchange Systems in the Aedes aegypti Mosquito and the Plutella xylostella Moth
Source: PLoS One. 2015 Apr 1;10(4):e0121097. doi: 10.1371/journal.pone.0121097 (PMC4382291; doi:10.1371/journal.pone.0121097)
Supplement: S1 File — (PDF) [file pone.0121097.s001.pdf]

Supplementary Figures

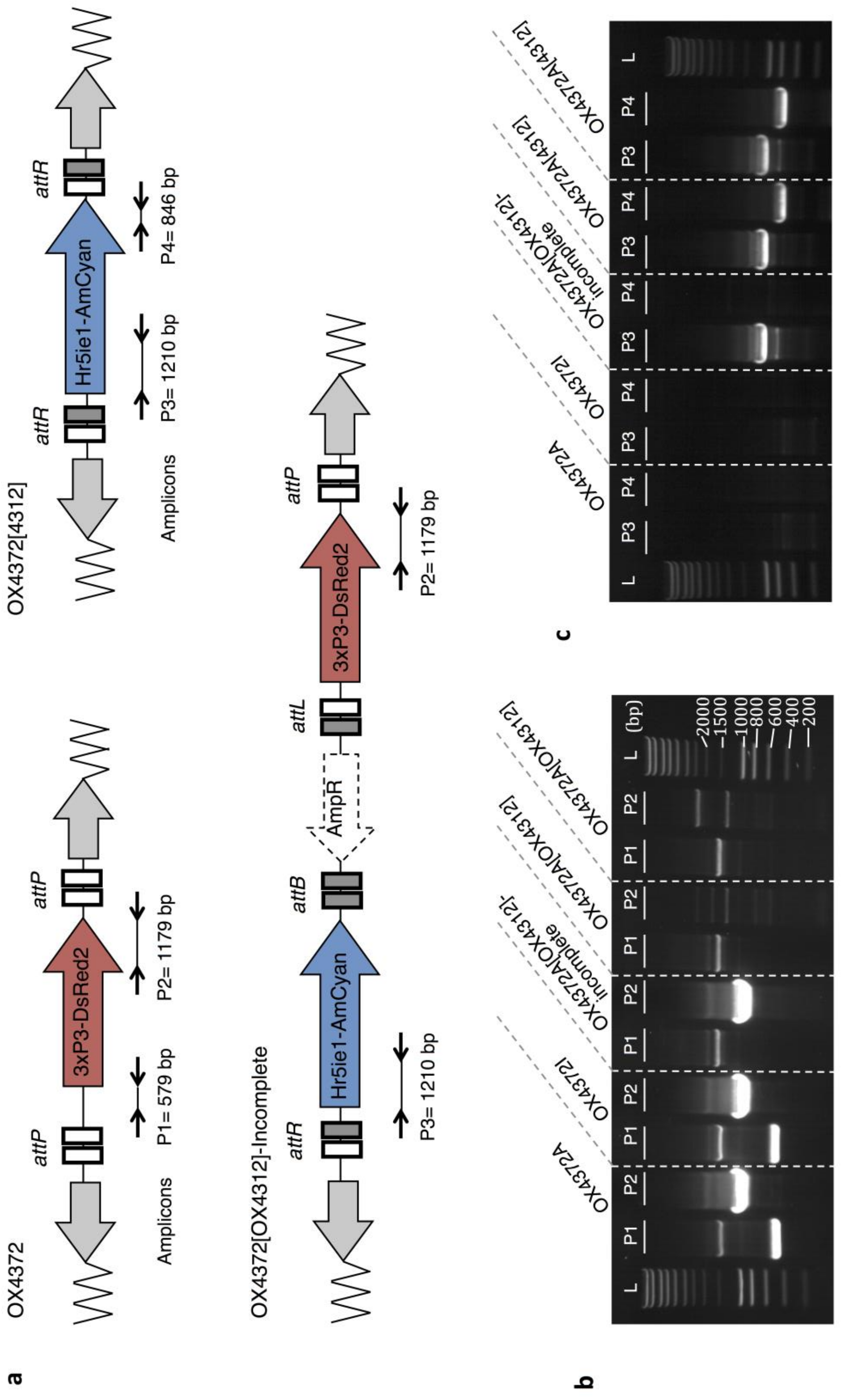

Figure-S1 (Haghighat-Khah)

Fig. A. ΦC31-RMCE in *Aedes aegypti.* (a) All situations are depicted with primers and amplification product sizes. PCR amplification between (b) the intact *attP*-DsRed junctions P1 and P2 in *Ae. aegypti* ΦC31-RMCE docking strains, and (c) the *attR*-AmCyan junctions P3 and P4 in strains following ΦC31-*att* recombination. Intact *attP*-DsRed 5' (P1) and 3' (P2) junctions were amplified in both docking lines OX4372A and OX4372I, but not in OX4372I[OX4312] and OX4372A[OX4312]. Recombined *attR*-AmCyan 5' (P3) and 3' (P4) junctions were amplified as expected in OX4372A[OX4312] and OX4372I[OX4312] indicating complete ΦC31-RMCE. In OX4372A[OX4312]-incomplete, the 3' *attP*-DsRed2 junction (P2) and 5' *attR*-AmCyan junction (P3) were amplified indicating recombination of only one set of ΦC31 attachment sites. The same DNA ladder was used in (b) and (c); L=DNA ladder (HyperLadder 1kb).

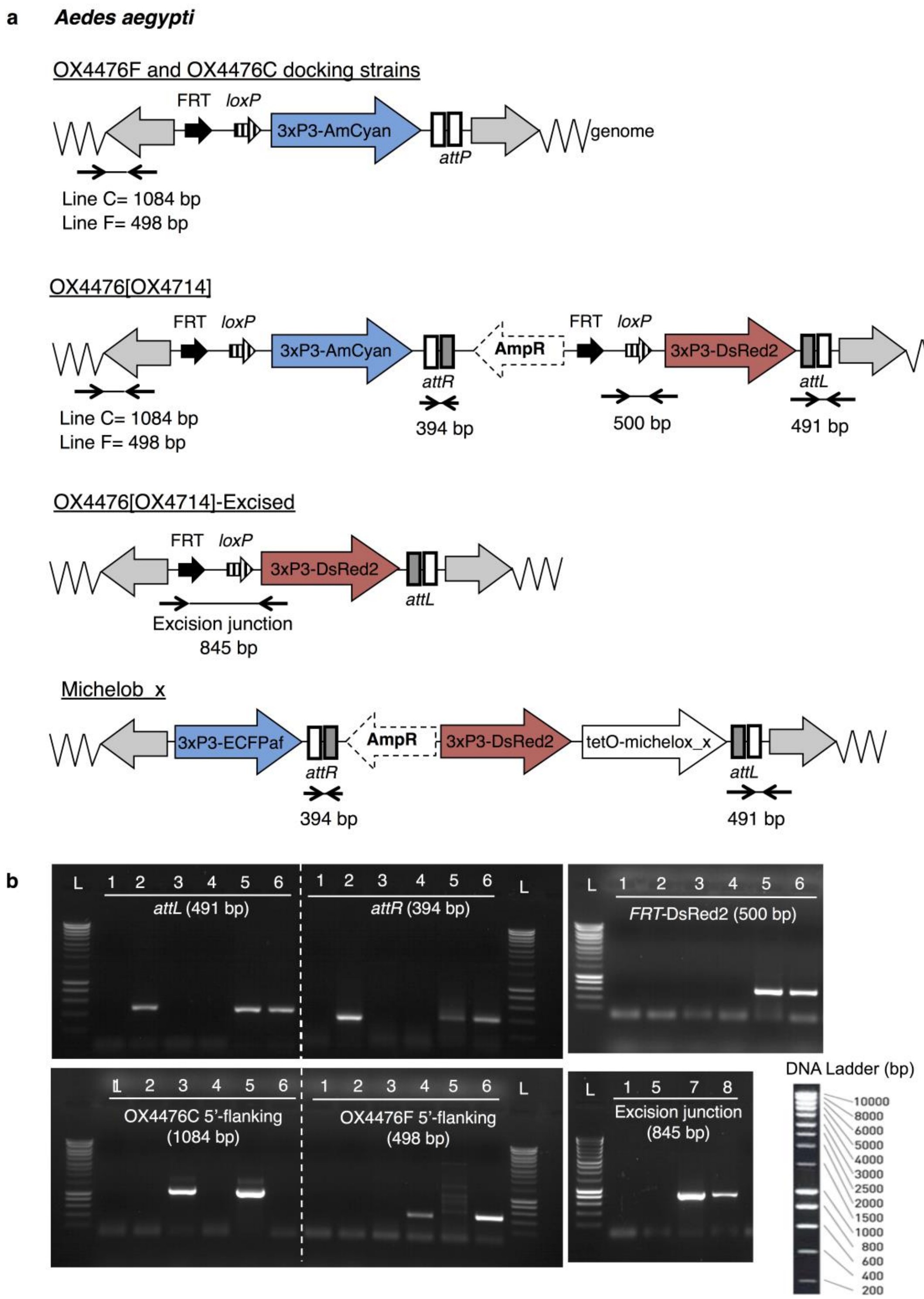

Fig. B. iRMCE in *Aedes aegypti.* (a) Schematic diagrams of strains used in (b) PCR analysis showing iRMCE between *Ae. aegypti* OX4476 docking transgenes and OX4714. Lane numbers refer to template DNA samples. Samples: 1 (H_2O) was a negative control for all primers; 2 (michelob_x; a different *Ae. aegypti* strain carrying a ΦC31-*att* integrated plasmid, described in Fu *et al*, 2010 (OX3582), Doi: 10.1073/pnas.1000251107) was a positive control for ΦC31-*att* integration (*attL* and *attR* amplicons); 3 (OX4476C) and 4 (OX4476F) were

negative controls for ΦC31-*att* integration (*attL* and *attR* amplicons), but positive controls for genomic flanking regions (OX4476C 5'-flanking and OX4476F 5'-flanking amplicons respectively). Canonical ΦC31-*att* integration was shown in DNA samples 5 (OX4476C[OX4714]) and 6 (OX4476F[OX4714]) due to the presence of the *attL* and *attR* amplicons and the donor cassette's *FRT*-DsRed2 amplicon; different OX4476 5'-flanking sequences were amplified in samples 5 and 6 indicating that these are two distinct iRMCE docking strains. Cre and FLP- mediated excision was indicated in samples 7 (OX4476F[OX4714] injected with Cre) and 8 (OX4476F[OX4714] injected with FLP) respectively because the expected 845 bp sequence across the excision junction was positively amplified. L= DNA Ladder (HyperLadder 1kb); the same DNA ladder was used for all gels.

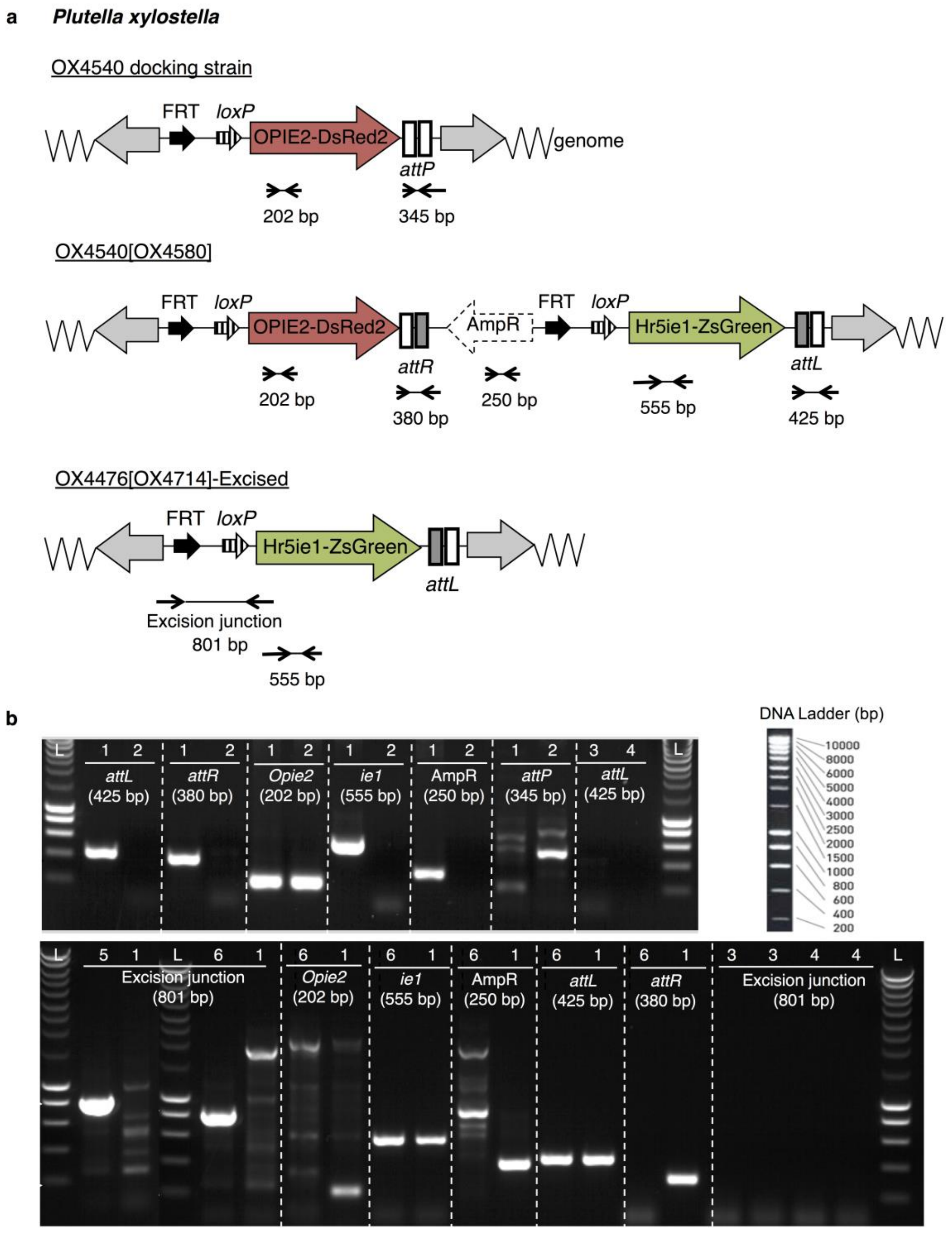

Figure-S3 (Haghighat-Khah)

Fig. C. iRMCE in *Plutella xylostella.* (a) Schematic diagrams of strains used in (b) PCR analysis showing iRMCE between *P. xylostella* OX4540 docking transgenes and OX4580**.** Lane numbers refer to template DNA samples. Samples: 3 (H_20) and 4 (wild-type) were negative controls for all primers; 2 (OX4540) was a negative control for ΦC31-*att* integration (*attL* and *attR* amplicons), but positive control for docking construct's *attP* and Opie2 transgenes. Canonical ΦC31-*att* integration was shown in sample 1 (OX4540[OX4580]) due to the presence of the *attL* and *attR* amplicons and the donor cassette's *ie1* and AmpR

amplicons, and the absence of the intact *attP* docking site. Cre and FLP- mediated excision was indicated in samples 5 (OX4580[OX4540] injected with Cre) and 6 (OX4580[OX4540] injected with FLP) respectively because the expected 801 bp sequence across the excision junction was positively amplified. L= DNA Ladder (HyperLadder 1kb); the same DNA ladder was used for all gels.

Supplementary Tables

Table A. Plasmid sequences

Plasmid	Sequence
OX4312	acaagttaaccgttgtaaaacgacggccagtgagctggccggcctaggcgcgccaagctt
	aaggtgcacggcccacgtggccactagtcgaggtcgacgatgtaggtcacggtctcgaag
	ccgcggtgcgggtgccagggcgtgcccttgggctccccgggcgcgtactccacctcaccc
	atctggtccatcatgatgaacgggtcgaggtggcggtagttgatcccggcgaacgcgcgg
	cgcaccgggaagccctcgccctcgaaaccgctgggcgcggtggtcacggtgagcacggga
	cgtgcgacggcgtcggcgggtgcggatacgcggggcagcgtcagcgggttctcgacggtc
	acggcgggcatgtcgacggtatcgataagcttgggcctcgagagggcccgccagctcgcc
	cggggatctaattcttaattaacactggtcggtccgagaaactcgcgttaagatacattg
	atgagtttggacaaaccacaactagaatgcagtgaaaaaaatgctttatttgtgaaattt
	gtgatgctattgctttatttgtaaccattataagctgcaataaacaagttaacaacaaca
	attgcattcattttatgtttcaggttcagggggaggtgtgggaggttttttaaagcaagt
	aaaacctctacaaatgtggtatggctgattatgatcagttatctagatccggtggatctt
	acgggtcctccaccttccgctttttcttgggtcgagatctgagtccggagaagggcacca
	cggaggtgatgtgggccacggcgtgctcggtcagctgcacgctgttgccgcccttgtcca
	ggtcggttctggcgatgcggtgctccaccacgtggttgggggcatggtcacgggcttct
	tggtcttgtaggaggtgtggaactggcatctgtagttgccgccgccctgcagcatcagga
	aggcggtcacgtcgcccttcaagatgccgtcgcacacggtcatcttctcgaaggaggggt
	cccagccggtggtcttcttggccatcacggggccgtcggcggggaagttcacgccgtgga
	aggtggacttgtgctcgaagcagttgcccttcaggctgatctcccagctggcggtggcca
	cgccgccgtcctcgtaggtgaaggttctctcgtaggacatgccgtcggggaaggcctgct
	tgaagtagtcgggcatgctggtggggtaggcggtgaagcagcggttgccgtacatgaaca
	cggtggacaggatgtcgaaggagaaggccagggggccgccgttggccatggtcaccttga
	aggtggaggtctgggtgccctcgtagggcttgccgctgccctcgcccttcacggtgaagt
	agtggccgttcacgcagccgtccatgtggtaggtcatcttcatgtcgtcgccgatgaact
	tgttggacagggccatggtggcgaccggtttgcgcttcttcttgggtggggtgggatctc
	ccatggtggcctgaatctcaacttgcacctgaaggtagtgcagcaaggatgagcaaaagg
	gaagaacccagaaaagaacgggaaaacttaccccaattagaattgcttgtcgccgccagt
	gtcaacttgcaactgaaacaatatccaacatgaacgtcaatttatactgccctaatggcg
	aacacgataacaatatttcttttattatgccctctaaaaccaacgcggttatcgtttatt
	tattcaaattagatatagaacatccgccgacatacaatgttaatgcaaaaacgcgtttgg
	tgagcggatacgaaaacagtcggccgataaacattaatctgaggtcgataacaccgtcct
	tgaacggaacacgaggagcgtacgtgatcagctgcattcgcgcgccgcgcctttatcgag
	atttatttgcatacaacaagtacactgcgccgttgggatttgtggtaacgcgcacacatg
	cagagctgcaagtgtggcacattttgtctgtgcgcaaaacctttgaagccaaaagtacga
	ggtccgttacgggcatgctagcgcacacggacaatggacccgacaaattctacgccaagg
	atttaatgataatgtcgggcaacgtatccgttcattttatcaataacctacaaaaatgtc
	gcgcgcatcacaaagacatcgatatatttaaacatttatgtcccgaactgcaaatcgata
	atagtgttgtgcaacctcgagcgtccgtttgatttaacgtatagcttgcaaatgaattat
	ttaattatcaatcatgttttacgcgtagaattctacccgtaaagcgagtttagttatgag
	ccatgtgcaaaacatgacatcagcttttatttttataacaaatgacatcatttcttgatt
	gtgttttacacgtagaattctactcgtaaagcgagttcagttttgaaaaacaaatgacat
	catcttttgattgtgctttacaagtagaattctacccgtaaatcaagttcggttttgaa
	aaacaaatgagtcatattgtatgatatcatattgcaaaacaaatgactcatcaatcgatc
	gtgcgttacacgtagaattctactcgtaaagcgagtttatgagccgtgtgcaaaacatga
	catcatctcgatttgaaaaacaaatgacatcatccactgatcgtgcattacaagtagaat
	tctactcgtaaagccagttcggttatgagccgtgtacaaaacatgacatcagattatgac
	tcatacttgattgtgttttacgcgtagaattctactcgtaaagccagttcaattttaaaa
	acaaatgacatcatccaaattaataaatgacaagcaatgggtacaaatctggccggccgc
	aaccattgtgggaaccggggccctctcgaggcccaagcttatcgataccgtcgacatgcc
	cgccgtgaccgtcgagaacccgctgacgctgccccgcgtatccgcacccgccgacgccgt
	cgcacgtcccgtgctcaccgtgaccaccgcgcccagcggtttcgagggcgagggcttccc
	ggtgcgccgcgcgttcgccgggatcaactaccgccacctcgacccgttcatcatgatgga

ccagatgggtgaggtggagtacgcgcccggggagcccaagggcacgccctggcacccgca
ccgcggcttcgagaccgtgacctacatcgtcgacctcgactagtggccacgtgggccgtg
caccttaagcttggcgcgcctaggccggccagctcactggccgtcgttttacaacgtcgt
gactgggaaaaccctggcgttacccaacttaatcgccttgcagcacatccccctttcgcc
agctggcgtaatagcgaagaggcccgcaccgatcgcccttcccaacagttgcgcagcctg
aatggcgaatggcgcctgatgcggtattttctccttacgcatctgtgcggtatttcacac
cgcatacgtcaaagcaaccatagtacgcgccctgtagcggcgcattaagcgcggcgggtg
tggtggttacgcgcagcgtgaccgctacacttgccagcgccctagcgcccgctcctttcg
ctttcttcccttcctttctcgccacgttcgccggctttccccgtcaagctctaaatcggg
ggctccctttagggttccgatttagtgctttacggcacctcgaccccaaaaaacttgatt
tgggtgatggttcacgtagtgggccatcgccctgatagacggtttttcgccctttgacgt
tggagtccacgttctttaatagtggactcttgttccaaactggaacaacactcaacccta
tctcgggctattcttttgatttataagggattttgccgatttcggcctattggttaaaaa
atgagctgatttaacaaaaatttaacgcgaattttaacaaaatattaacgtttacaattt
tatggtgcactctcagtacaatctgctctgatgccgcatagttaagccagccccgacacc
cgccaacacccgctgacgcgccctgacgggcttgtctgctcccggcatccgcttacagac
aagctgtgaccgtctccgggagctgcatgtgtcagaggttttcaccgtcatcaccgaaac
gcgcgagacgaaagggcctcgtgatacgcctatttttataggttaatgtcatgataataa
tggtttcttagacgtcaggtggcacttttcggggaaatgtgcgcggaacccctatttgtt
tatttttctaaatacattcaaatatgtatccgctcatgagacaataaccctgataaatgc
ttcaataatattgaaaaaggaagagtatgagtattcaacatttccgtgtcgcccttattc
ccttttttgcggcattttgccttcctgtttttgctcacccagaaacgctggtgaaagtaa
aagatgctgaagatcagttgggtgcacgagtgggttacatcgaactggatctcaacagcg
gtaagatccttgagagttttcgccccgaagaacgttttccaatgatgagcacttttaaag
ttctgctatgtggcgcggtattatcccgtattgacgccgggcaagagcaactcggtcgcc
gcatacactattctcagaatgacttggttgagtactcaccagtcacagaaaagcatctta
cggatggcatgacagtaagagaattatgcagtgctgccataaccatgagtgataacactg
cggccaacttacttctgacaacgatcggaggaccgaaggagctaaccgcttttttgcaca
acatgggggatcatgtaactcgccttgatcgttgggaaccggagctgaatgaagccatac
caaacgacgagcgtgacaccacgatgcctgtagcaatggcaacaacgttgcgcaaactat
taactggcgaactacttactctagcttcccggcaacaattaatagactggatggaggcgg
ataaagttgcaggaccacttctgcgctcggcccttccggctggctggtttattgctgata
aatctggagccggtgagcgtgggtctcgcggtatcattgcagcactggggccagatggta
agccctcccgtatcgtagttatctacacgacggggagtcaggcaactatggatgaacgaa
atagacagatcgctgagataggtgcctcactgattaagcattggtaactgtcagaccaag
tttactcatatatactttagattgatttaaaacttcatttttaatttaaaaggatctagg
tgaagatcctttttgataatctcatgaccaaaatcccttaacgtgagttttcgttccact
gagcgtcagaccccgtagaaaagatcaaaggatcttcttgagatcctttttttctgcgcg
taatctgctgcttgcaaacaaaaaaaccaccgctaccagcggtggtttgtttgccggatc
aagagctaccaactctttttccgaaggtaactggcttcagcagagcgcagataccaaata
ctgttcttctagtgtagccgtagttaggccaccacttcaagaactctgtagcaccgccta
catacctcgctctgctaatcctgttaccagtggctgctgccagtggcgataagtcgtgtc
ttaccgggttggactcaagacgatagttaccggataaggcgcagcggtcgggctgaacgg
ggggttcgtgcacacagcccagcttggagcgaacgacctacaccgaactgagatacctac
agcgtgagctatgagaaagcgccacgcttcccgaagggagaaaggcggacaggtatccgg
taagcggcagggtcggaacaggagagcgcacgagggagcttccagggggaaacgcctggt
atctttatagtcctgtcgggtttcgccacctctgacttgagcgtcgatttttgtgatgct
cgtcaggggggcggagcctatggaaaaacgccagcaacgcggcctttttacggttcctgg
ccttttgctggccttttgctcacatgttctttcctgcgttatcccctgattctgtggata
accgtattaccgcctttgagtgagctgataccgctcgccgcagccgaacgaccgagcgca
gcgagtcagtgagcgaggaagcggaagagcgcccaatacgcaaaccgcctctccccgcgc
gttggccgattcattaatgcagctggcacgacaggtttcccgactggaaagcgggcagtg
agcgcaacgcaattaatgtgagttagctcactcattaggcaccccaggctttacacttta
tgcttccggctcgtatgttgtgtggaattgtgagcggataacaatttcacacaggaaaca
gctatgaccatgattaggcgcgcctaggccggccgaattcgaatggccatgtaagataca
ttgatgagtttggacaaaccacaactagaatgcagtgaaaaaaatgctttatttgtgaaa
tttgtgatgctattgctttatttgtaaccattataagctgcaataa

OX4372
ccccctcgaggttcccacaatggttaattcgagctcgcccggggatctaattcaattaga
gactaattcaattagagctaattcaattaggatccaagcttatcgatttcgaaccctcga
ccgccggagtataaatagaggcgcttcgtctacggagcgacaattcaattcaaacaagca

aagtgaacacgtcgctaagcgaaagctaagcaaataaacaagcgcagctgaacaagctaa
acaatcggggtaccgctagagtcgatcccaccccacccaagaagaagcgcaaaccggtac
catggcctcctccgagaacgtcatcaccgagttcatgcgcttcaaggtgcgcatggaggg
caccgtgaacggccacgagttcgagatcgagggcgagggcgagggccgcccctacgaggg
ccacaacaccgtgaagctgaaggtgaccaagggcggcccccctgcccttcgcctgggacat
cctgtcccccccagttccagtacggctccaaggtgtacgtgaagcaccccgccgacatccc
cgactacaagaagctgtccttccccgagggcttcaagtgggagcgcgtgatgaacttcga
ggacggcggcgtggcgaccgtgacccaggactcctccctgcaggacggctgcttcatcta
caaggtgaagttcatcggcgtgaacttcccctccgacggccccgtgatgcagaagaagac
catgggctgggaggcctccaccgagcgcctgtaccccccgcgacggcgtgctgaagggcga
gacccacaaggccctgaagctgaaggacggcggccactacctggtggagttcaagtccat
ctacatggccaagaagcccgtgcagctgcccggctactactacgtggacgccaagctgga
catcacctcccacaacgaggactacaccatcgtggagcagtacgagcgcaccgagggccg
ccaccacctgttcctgtgatgatcataatcagccataccacatttgtagaggttttactt
gctttaaaaaacctcccacacctccccctgaacctgaaacataaaatgaatgcaattgtt
gttgttaacttgtttattgcagcttataatggttacaaataaagcaatagcatcacaaat
ttcacaaataaagcatttttttcactgcattctagttgtggtttgtccaaactcatcaat
gtatcttaacgcgagttaattaaggccgctcatttttaaggtgcacggcccacgtggcca
ctagctcgcgctcgcgcgactgacggtcgtaagcacccgcgtacgtgtccaccccggtca
caaccccttgtgtcatgtcggcgaccctacgcccccaactgagagaactcaaaggttacc
ccagttggggcactactcccgaaaaccgcttctgacctgggaaaacgtgaagccccgggg
catccgctgagggttgccgccggggcttcggtgtgtccgtcagtacttaataaatctggc
cggccgcctgcagtaggaagacgaataggtggcctatggcattattgtacggaatgataa
acattgcctgcataaattcttttattatatacagccataatgtcagtagcaagggagaaa
aggtccaaagtcgcaaaaaatttatgagaaacctttacatgagcctgacgtcatcgttta
tgcgtaagcgtttagaagctcctactttgaagagatatttgcgcgataatatctctaata
ttttgccaaatgaagtgcctggtacatcagatgacagtactgaagagccagtaatgaaaa
aacgtacttactgtacttactgcccctctaaaataaggcgaaaggcaaatgcatcgtgca
aaaaatgcaaaaaagttatttgtcgagagcataatattgatatgtgccaaagttgtttct
gactgactaataagtataatttgtttctattatgtataagttaagctaattacttatttt
ataatacaacatgactgtttttaaagtacaaaataagtttatttttgtaaaagagagaat
gtttaaaagttttgttactttatagaagaaattttgagtttttgtttttttttaataaat
aaataaacataaataaattgtttgttgaatttattattagtatgtaagtgtaaatataat
aaaacttaatatctattcaaattaataaataaacctcgatatacagaccgataaaacaca
tgcgtcaattttacgcatgattatctttaacgtacgtcacaatatgattatctttctagg
gttaaataatagtttctaattttttttattattcagcctgctgtcgtgaataccgtatatc
tcaacgctgtctgtgagattgtcgtattctagcctttttagtttttcgctcatcgacttg
atattgtccgacacattttcgtcgatttgcgttttgatcaaagacttgagcagagacacg
ttaatcaactgttcaaattgatccatattaacgatatcaacccgatgcgtatatggtgcg
taaaatatatttttttaaccctcttatactttgcactctgcgttaatacgcgttcgtgtac
agacgtaatcatgttttctttttttggataaaactcctactgagtttgacctcatattaga
ccctcacaagttgcaaaacgtggcatttttttaccaatgaagaatttaaagttattttaaa
aaatttcatcacagatttaaagaagaaccaaaaattaaattatttcaacagtttaatcga
ccagttaatcaacgtgtacacagacgcgtcggcaaaaaacacgcagcccgacgtgttggc
taaaattattaaatcaacttgtgttatagtcacggatttgccgtccaacgtgttcctcaa
aaagttgaagaccaacaagtttacggacactattaattatttgattttgccccacttcat
tttgtgggatcacaattttgttatattttaaacaaagcttggcactggccgtcgttttac
aacgtcgtgactgggaaaaccctggcgttacccaacttaatcgccttgcagcacatcccc
ctttcgccagctggcgtaatagcgaagaggcccgcaccgatcgcccttcccaacagttgc
gcagcctgaatggcgaatggcgcctgatgcggtattttctccttacgcatctgtgcggta
tttcacaccgcatatggtgcactctcagtacaatctgctctgatgccgcatagttaagcc
agccccgacacccgccaacacccgctgacgcgccctgacgggcttgtctgctcccggcat
ccgcttacagacaagctgtgaccgtctccgggagctgcatgtgtcagaggttttcaccgt
catcaccgaaacgcgcgagacgaaagggcctcgtgatacgcctatttttataggttaatg
tcatgataataatggtttcttagacgtcaggtggcacttttcggggaaatgtgcgcggaa
cccctatttgtttatttttctaaatacattcaaatatgtatccgctcatgagacaataac
cctgataaatgcttcaataatattgaaaaaggaagagtatgagtattcaacatttccgtg
tcgcccttattcccttttttgcggcattttgccttcctgtttttgctcacccagaaacgc
tggtgaaagtaaaagatgctgaagatcagttgggtgcacgagtgggttacatcgaactgg
atctcaacagcggtaagatccttgagagttttcgccccgaagaacgttttccaatgatga
gcacttttaaagttctgctatgtggcgcggtattatcccgtattgacgccgggcaagagc

aactcggtcgccgcatacactattctcagaatgacttggttgagtactcaccagtcacag<br>aaaagcatcttacggatggcatgacagtaagagaattatgcagtgctgccataaccatga<br>gtgataacactgcggccaacttacttctgacaacgatcggaggaccgaaggagctaaccg<br>ctttttgcacaacatgggggatcatgtaactcgccttgatcgttgggaaccggagctga<br>atgaagccataccaaacgacgagcgtgacaccacgatgcctgtagcaatggcaacaacgt<br>tgcgcaaactattaactggcgaactacttactctagcttcccggcaacaattaatagact<br>ggatggaggcggataaagttgcaggaccacttctgcgctcggcccttccggctggctggt<br>ttattgctgataaatctggagccggtgagcgtgggtctcgcggtatcattgcagcactgg<br>ggccagatggtaagccctcccgtatcgtagttatctacacgacggggagtcaggcaacta<br>tggatgaacgaaatagacagatcgctgagataggtgcctcactgattaagcattggtaac<br>tgtcagaccaagtttactcatatatactttagattgatttaaaacttcatttttaattta<br>aaaggatctaggtgaagatcctttttgataatctcatgaccaaaatcccttaacgtgagt<br>tttcgttccactgagcgtcagaccccgtagaaaagatcaaaggatcttcttgagatcctt<br>tttttctgcgcgtaatctgctgcttgcaaacaaaaaaaccaccgctaccagcggtggttt<br>gtttgccggatcaagagctaccaactctttttccgaaggtaactggcttcagcagagcgc<br>agataccaaatactgtccttctagtgtagccgtagttaggccaccacttcaagaactctg<br>tagcaccgcctacatacctcgctctgctaatcctgttaccagtggctgctgccagtggcg<br>ataagtcgtgtcttaccgggttggactcaagacgatagttaccggataaggcgcagcggt<br>cgggctgaacggggggttcgtgcacacagcccagcttggagcgaacgacctacaccgaac<br>tgagatacctacagcgtgagcattgagaaagcgccacgcttcccgaagggagaaaggcgg<br>acaggtatccggtaagcggcagggtcggaacaggagagcgcacgagggagcttccagggg<br>gaaacgcctggtatctttatagtcctgtcgggtttcgccacctctgacttgagcgtcgat<br>ttttgtgatgctcgtcaggggggcggagcctatggaaaaacgccagcaacgcggcctttt<br>tacggttcctggccttttgctggccttttgctcacatgttctttcctgcgttatcccctg<br>attctgtggataaccgtattaccgcctttgagtgagctgataccgctcgccgcagccgaa<br>cgaccgagcgcagcgagtcagtgagcgaggaagcggaagagcgcccaatacgcaaaccgc<br>ctctccccgcgcgttggccgattcattaatgcagctggcacgacaggtttcccgactgga<br>aagcgggcagtgagcgcaacgcaattaatgtgagttagctcactcattaggcaccccagg<br>ctttacactttatgcttccggctcgtatgttgtgtggaattgtgagcggataacaatttc<br>acacaggaaacagctatgaccatgattacgaattcgagctcggtacccggggatcctcta<br>gagtcgacgctcgcgcgacttggtttgccattctttagcgcgcgtcgcgtcacacagctt<br>ggccacaatgtggtttttgtcaaacgaagattctatgacgtgtttaaagtttaggtcgag<br>taaagcgcaaatctttttttaaccctagaaagatagtctgcgtaaaattgacgcatgcatt<br>cttgaaatattgctctctctttctaaatagcgcgaatccgtcgctgtgcatttaggacat<br>ctcagtcgccgcttggagctcccgtgaggcgtgcttgtcaatgcggtaagtgtcactgat<br>tttgaactataacgaccgcgtgagtcaaaatgacgcatgattatcttttacgtgactttt<br>aagatttaactcatacgataattatattgttatttcatgttctacttacgtgataactta<br>ttatatatatatttcttgttatagatatcgtgactaatatataataaaatgggtagttc<br>tttagacgatgagcatatcctctctgctcttctgcaaagcgatgacgagcttgttggtga<br>ggattctgacagtgaaatatcagatcacgtaagtgaagatgacgtccagagcgatacaga<br>agaagcgtttatagatgaggtacatgaagtgcagccaacgtcaagcggtagtgaaatatt<br>agacgaacaaaatgttattgaacaaccaggttcttcattggcttctaacagaatcttgac<br>cttgccacagaggactattagaggtaagaataaacattgttggtcaacttcaaagtccac<br>gaggcgtagccgagtctctgcactgaacattgtcagatctcaaagaggtccgacgcgtat<br>gtgccgcaatatatatgacccacttttatgcttcaaactatttttactgatgagataat<br>ttcggaaattgtaaaatggacaaatgctgagatatcattgaaacgtcgggaatctatgac<br>aggtgctacatttcgtgacacgaatgaagatgaaatctatgctttctttggtattctggt<br>aatgacagcagtgagaaaagataaccacatgtccacagatgacctctttgatcgatcttt<br>gtcaatggtgtacgtctctgtaatgagtcgtgatcgttttgatttttttgatacgatgtct<br>tagaatggatgacaaaagtatacggcccacacttcgagaaaacgatgtatttactcctgt<br>tagaaaaatatgggatctctttatccatcagtgcatacaaaattacactccaggggctca<br>tttgaccatagatgaacagttacttggttttagaggacggtgtccgtttaggatgtatat<br>cccaaacaagccaagtaagtatggaataaaaatcctcatgatgtgtgacagtggtacgaa<br>gtatatgataaatggaatgccttatttgggaagaggaacacagaccaacggagtaccact<br>cggtgaatactacgtgaaggagttatcaaagcctgtgcacggtagttgtcgtaatattac<br>gtgtgacaattggttcacctcaatccctttggcaaaaaacttactacaagaaccgtataa<br>gttcgagatcggctaggtgtcgacggtatcgataagcttgggccattaagtactgacgga<br>cacaccgaagccccggcggcaaccctcagcggatgccccggggcttcacgttttcccagg<br>tcagaagcggttttcgggagtagtgccccaactggggtaacctttgagttctctcagttg<br>gggcgtagggtcgccgacatgacacaaggggttgtgaccggggtggacacgtacgcggg<br>tgcttacgaccgtcagtcgcgcgagcgcgagctagtggccacgtgggccgtgcaccttaa

OX4476

tggagccggtgagcgtgggtctcgcggtatcattgcagcactggggccagatggtaagcc
ctcccgtatcgtagttatctacacgacggggagtcaggcaactatggatgaacgaaatag
acagatcgctgagataggtgcctcactgattaagcattggtaactgtcagaccaagttta
ctcatatatactttagattgatttaaaacttcatttttaatttaaaaggatctaggtgaa
gatcctttttgataatctcatgaccaaaatcccttaacgtgagttttcgttccactgagc
gtcagaccccgtagaaaagatcaaaggatcttcttgagatcctttttttctgcgcgtaat
ctgctgcttgcaaacaaaaaaaccaccgctaccagcggtggtttgtttgccggatcaaga
gctaccaactctttttccgaaggtaactggcttcagcagagcgcagataccaaatactgt
ccttctagtgtagccgtagttaggccaccacttcaagaactctgtagcaccgcctacata
cctcgctctgctaatcctgttaccagtggctgctgccagtggcgataagtcgtgtcttac
cgggttggactcaagacgatagttaccggataaggcgcagcggtcgggctgaacgggggg
ttcgtgcacacagcccagcttggagcgaacgacctacaccgaactgagatacctacagcg
tgagcattgagaaagcgccacgcttcccgaagggagaaaggcggacaggtatccggtaag
cggcagggtcggaacaggagagcgcacgagggagcttccagggggaaacgcctggtatct
ttatagtcctgtcgggtttcgccacctctgacttgagcgtcgatttttgtgatgctcgtc
aggggggcggagcctatggaaaaacgccagcaacgcggcctttttacggttcctggcctt
ttgctggccttttgctcacatgttctttcctgcgttatcccctgattctgtggataaccg
tattaccgcctttgagtgagctgataccgctcgccgcagccgaacgaccgagcgcagcga
gtcagtgagcgaggaagcggaagagcgcccaatacgcaaaccgcctctccccgcgcgttg
gccgattcattaatgcagctggcacgacaggtttcccgactggaaagcgggcagtgagcg
caacgcaattaatgtgagttagctcactcattaggcaccccaggctttacactttatgct
tccggctcgtatgttgtgtggaattgtgagcggataacaatttcacacaggaaacagcta
tgaccatgattacgaattcgagctcggtacccggggatcctctagagtcgacgctcgcgc
gacttggtttgccattctttagcgcgcgtcgcgtcacacagcttggccacaatgtggttt
ttgtcaaacgaagattctatgacgtgtttaaagtttaggtcgagtaaagcgcaaatcttt
tttaaccctagaaagatagtctgcgtaaaattgacgcatgcattcttgaaatattgctct
ctctttctaaatagcgcgaatccgtcgctgtgcatttaggacatctcagtcgccgcttgg
agctcccgtgaggcgtgcttgtcaatgcggtaagtgtcactgattttgaactataacgac
cgcgtgagtcaaaatgacgcatgattatcttttacgtgacttttaagatttaactcatac
gataattatattgttatttcatgttctacttacgtgataacttattatatatatatttc
ttgttatagatatcgtgactaatatataataaaatgggtagttctttagacgatgagcat
atcctctctgctcttctgcaaagcgatgacgagcttgttggtgaggattctgacagtgaa
atatcagatcacgtaagtgaagatgacgtccagagcgatacagaagaagcgtttatagat
gaggtacatgaagtgcagccaacgtcaagcggtagtgaaatattagacgaacaaaatgtt
attgaacaaccaggttcttcattggcttctaacagaatcttgaccttgccacagaggact
attagaggtaagaataaacattgttggtcaacttcaaagtccacgaggcgtagccgagtc
tctgcactgaacattgtcagatctcaaagaggtccgacgcgtatgtgccgcaatatatat
gacccacttttatgcttcaaactatttttactgatgagataatttcggaaattgtaaaa
tggacaaatgctgagatatcattgaaacgtcgggaatctatgacaggtgctacatttcgt
gacacgaatgaagatgaaatctatgctttctttggtattctggtaatgacagcagtgaga
aaagataaccacatgtccacagatgacctctttgatcgatctttgtcaatggtgtacgtc
tctgtaatgagtcgtgatcgttttgatttttttgatacgatgtcttagaatggatgacaaa
agtatacggcccacacttcgagaaaacgatgtatttactcctgttagaaaaatatgggat
ctctttatccatcagtgcatacaaaattacactccaggggctcatttgaccatagatgaa
cagttacttggttttagaggacggtgtccgtttaggatgtatatcccaaacaagccaagt
aagtatggaataaaaatcctcatgatgtgtgacagtggtacgaagtatatgataaatgga
atgccttatttgggaagaggaacacagaccaacggagtaccactcggtgaatactacgtg
aaggagttatcaaagcctgtgcacggtagttgtcgtaatattacgtgtgacaattggttc
acctcaatccctttggcaaaaaacttactacaagaaccgtataagttcgagatcggccgg
cctagctcgaagaagttcctattccgaagttcctattctctagaaagtataggaacttcc
ctaggataacttcgtataatgtatgctatacgaagttatcctgcggcgcgccatgcggcc
gcgcgttaagatacattgatgagtttggacaaaccacaactagaatgcagtgaaaaaaat
gctttatttgtgaaatttgtgatgctattgctttatttgtaaccattataagctgcaata
aacaagttaacaacaacaattgcattcattttatgtttcaggttcagggggaggtgtggg
aggtttttttaaagcaagtaaaacctctacaaatgtggtatggctgattatgatctgagtc
cggagaagggcaccacggaggtgatgtgggccacggcgtgctcggtcagctgcacgctgt
tgccgcccttgtccaggtcggttctggcgatgcggtgctccaccacgtggttgggggca
tggtcacgggcttcttggtcttgtaggaggtgtggaactggcatctgtagttgccgccgc
cctgcagcatcaggaaggcggtcacgtcgcccttcaagatgccgtcgcacacggtcatct
tctcgaaggaggggtcccagccggtggtcttcttggccatcacggggccgtcggcgggga

agttcacgccgtggaaggtggacttgtgctcgaagcagttgcccttcaggctgatctccc
agctggcggtggccacgccgccgtcctcgtaggtgaaggttctctcgtaggacatgccgt
cggggaaggcctgcttgaagtagtcgggcatgctggtggggtaggcggtgaagcagcggt
tgccgtacatgaacacggtggacaggatgtcgaaggagaaggccaggggccgccgttgg
ccatggtcaccttgaaggtggaggtctgggtgccctcgtagggcttgccgctgccctcgc
ccttcacggtgaagtagtggccgttcacgcagccgtccatgtggtaggtcatcttcatgt
cgtcgccgatgaacttgttggacagggccatggtggcgtcatggtggcgaccggtggatc
gtaccgtcgactctagcggtaccccgattgtttagcttgttcagctgcgcttgtttattt
gcttagctttcgcttagcgacgtgttcactttgcttgtttgaattgaattgtcgctccgt
agacgaagcgcctctatttatactccggcggtcgagggttcgaaatcgataagcttggat
cctaattgaattagctctaattgaattagtctctaattgaattagatccccgggcgagct
cgaattaaccattgtgggaaccgtgcgatcaaacaaacgcgagataccggaagtactgaa
aaacagtcgctccaggccagtgggaacatcgatgttttgttttgacggaccccttactct
cgtctcatataaaccgaagccagctaagatggtatacttattatcatcttgtgatgagga
tgcttctatcaacgaaagtaccggtaaaccgcaaatggttatgtattataatcaaactaa
aggcggagtggacacgctagaccaaatgtgttctgtgatgacctgcagcccggggatcc
actagtactgacggacacaccgaagccccggcggcaaccctcagcggatgccccggggct
tcacgttttcccaggtcagaagcggttttcgggagtagtgccccaactggggtaaccttt
gagttctctcagttgggggcgtagggccgccgacatgacacaaggggttgtgaccggggt
ggacacgtacgcgggtgcttacgaccgtcagtcgcgcgagcgcgactagttctagagcgg
ccgcctgcagtaggaagacgaataggtggcctatggcattattgtacggaatgataaaca
ttgcctgcataaattcttttattatatacagccataatgtcagtagcaagggagaaaagg
tccaaagtcgcaaaaaatttatgagaaacctttacatgagcctgacgtcatcgtttatgc
gtaagcgtttagaagctcctactttgaagagatatttgcgcgataatatctctaatattt
tgccaaatgaagtgcctggtacatcagatgacagtactgaagagccagtaatgaaaaaac
gtacttactgtacttactgcccctctaaaataaggcgaaaggcaaatgcatcgtgcaaaa
aatgcaaaaaagttatttgtcgagagcataatattgatatgtgccaaagttgtttctgac
tgactaataagtataatttgtttctattatgtataagttaagctaattacttattttata
atacaacatgactgtttttaaagtacaaaataagtttatttttgtaaaagagagaatgtt
taaaagttttgttactttatagaagaaattttgagtttttgtttttttttaataaataaa
taaacataaataaattgtttgttgaatttattattagtatgtaagtgtaaatataataaa
acttaatatctattcaaattaataaataaacctcgatatacagaccgataaaacacatgc
gtcaattttacgcatgattatctttaacgtacgtcacaatatgattatctttctagggtt
aaataatagtttctaatttttttattattcagcctgctgtcgtgaataccgtatatctca
acgctgtctgtgagattgtcgtattctagcctttttagtttttcgctcatcgacttgata
ttgtccgacacattttcgtcgatttgcgttttgatcaaagacttgagcagagacacgtta
atcaactgttcaaattgatccatattaacgatatcaacccgatgcgtatatggtgcgtaa
aatatatttttaaccctcttatactttgcactctgcgttaatacgcgttcgtgtacaga
cgtaatcatgtttctttttggataaaactcctactgagtttgacctcatattagaccc
tcacaagttgcaaaacgtggcatttttaccaatgaagaatttaaagttattttaaaaaa
tttcatcacagatttaaagaagaaccaaaaattaaattatttcaacagtttaatcgacca
gttaatcaacgtgtacacagacgcgtcggcaaaaaacacgcagcccgacgtgttggctaa
aattattaaatcaacttgtgttatagtcacggatttgccgtccaacgtgttcctcaaaaa
gttgaagaccaacaagtttacggacactattaattatttgattttgccccacttcatttt
gtgggatcacaattttgttatattttaaacaaagcttggcactggccgtcgttttacaac
gtcgtgactgggaaaaccctggcgttacccaacttaatcgccttgcagcacatccccctt
tcgccagctggcgtaatagcgaagaggcccgcaccgatcgcccttcccaacagttgcgca
gcctgaatggcgaatggcgcctgatgcggtattttctccttacgcatctgtgcggtattt
cacaccgcatatggtgcactctcagtacaatctgctctgatgccgcatagttaagccagc
cccgacacccgccaacacccgctgacgcgccctgacgggcttgtctgctcccggcatccg
cttacagacaagctgtgaccgtctccgggagctgcatgtgtcagaggttttcaccgtcat
caccgaaacgcgcgagacgaaagggcctcgtgatacgcctatttttataggttaatgtca
tgataataatggtttcttagacgtcaggtggcacttttcggggaaatgtgcgcggaaccc
ctatttgtttatttttctaaatacattcaaatatgtatccgctcatgagacaataaccct
gataaatgcttcaataatattgaaaaaggaagagtatgagtattcaacatttccgtgtcg
cccttattcccttttttgcggcattttgccttcctgtttttgctcacccagaaacgctgg
tgaaagtaaaagatgctgaagatcagttgggtgcacgagtgggttacatcgaactggatc
tcaacagcggtaagatccttgagagttttcgccccgaagaacgttttccaatgatgagca
cttttaaagttctgctatgtggcgcggtattatcccgtattgacgccgggcaagagcaac
tcggtcgccgcatacactattctcagaatgacttggttgagtactcaccagtcacagaaa
agcatcttacggatggcatgacagtaagagaattatgcagtgctgccataaccatgagtg

ataacactgcggccaacttacttctgacaacgatcggaggaccgaaggagctaaccgctt
ttttgcacaacatgggggatcatgtaactcgccttgatcgttgggaaccggagctgaatg
aagccataccaaacgacgagcgtgacaccacgatgcctgtagcaatggcaacaacgttgc
gcaaactattaactggcgaactacttactctagcttcccggcaacaattaatagactgga
tggaggcggataaagttgcaggaccacttctgcgctcggcccttccggctggctggttta
ttgctgataaatc

OX4540

cgaaatcgataagcttggatcctaattgaattagctctaattgaattagtctctaattga
attagatccccgggcgagctcgaattaaccattgtgggaaccgtgcgatcaaacaaacgc
gagataccggaagtactgaaaaacagtcgctccaggccagtgggaacatcgatgttttgt
tttgacggaccccttactctcgtctcatataaaccgaagccagctaagatggtatactta
ttatcatcttgtgatgaggatgcttctatcaacgaaagtaccggtaaaccgcaaatggtt
atgtattataatcaaactaaaggcggagtggacacgctagaccaaatgtgttctgtgatg
acctgcagcccgggggatccactagtactgacggacacaccgaagccccggcggcaaccc
tcagcggatgccccggggcttcacgttttcccaggtcagaagcggttttcgggagtagtg
ccccaactggggtaacctttgagttctctcagttgggggcgtagggccgccgacatgaca
caaggggttgtgaccggggtggacacgtacgcgggtgcttacgaccgtcagtcgcgcgag
cgcgactagttctagagcggccgcctgcagtaggaagacgaataggtggcctatggcatt
attgtacggaatgataaacattgcctgcataaattcttttattatatacagccataatgt
cagtagcaagggagaaaaggtccaaagtcgcaaaaaatttatgagaaacctttacatgag
cctgacgtcatcgtttatgcgtaagcgtttagaagctcctactttgaagagatatttgcg
cgataatatctctaatattttgccaaatgaagtgcctggtacatcagatgacagtactga
agagccagtaatgaaaaaacgtacttactgtacttactgcccctctaaaataaggcgaaa
ggcaaatgcatcgtgcaaaaaatgcaaaaaagttatttgtcgagagcataatattgatat
gtgccaaagttgtttctgactgactaataagtataatttgtttctattatgtataagtta
agctaattacttattttataatacaacatgactgtttttaaagtacaaaataagtttatt
tttgtaaaagagagaatgtttaaaagttttgttactttatagaagaaattttgagttttt
gtttttttttaataaataaataaacataaataaattgtttgttgaatttattattagtat
gtaagtgtaaatataataaaacttaatatctattcaaattaataaataaacctcgatata
cagaccgataaaacacatgcgtcaattttacgcatgattatctttaacgtacgtcacaat
atgattatctttctagggttaaataatagtttctaatttttttattattcagcctgctgt
cgtgaataccgtatatctcaacgctgtctgtgagattgtcgtattctagccttttttagtt
tttcgctcatcgacttgatattgtccgacacattttcgtcgatttgcgttttgatcaaag
acttgagcagagacacgttaatcaactgttcaaattgatccatattaacgatatcaaccc
gatgcgtatatggtgcgtaaaatatatttttttaaccctcttatactttgcactctgcgtt
aatacgcgttcgtgtacagacgtaatcatgtttttctttttttggataaaactcctactgag
tttgacctcatattagaccctcacaagttgcaaaacgtggcatttttttaccaatgaagaa
tttaaagttattttaaaaaatttcatcacagatttaaagaagaaccaaaaattaaattat
ttcaacagtttaatcgaccagttaatcaacgtgtacacagacgcgtcggcaaaaaacacg
cagcccgacgtgttggctaaaattattaaatcaacttgtgttatagtcacggatttgccg
tccaacgtgttcctcaaaaagttgaagaccaacaagtttacggacactattaattatttg
attttgccccacttcattttgtgggatcacaattttgttatattttaaacaaagcttggc
actggccgtcgttttacaacgtcgtgactgggaaaaccctggcgttacccaacttaatcg
ccttgcagcacatccccctttcgccagctggcgtaatagcgaagaggcccgcaccgatcg
cccttcccaacagttgcgcagcctgaatggcgaatggcgcctgatgcggtattttctcct
tacgcatctgtgcggtatttcacaccgcatatggtgcactctcagtacaatctgctctga
tgccgcatagttaagccagccccgacacccgccaacacccgctgacgcgccctgacgggc
ttgtctgctcccggcatccgcttacagacaagctgtgaccgtctccgggagctgcatgtg
tcagaggttttcaccgtcatcaccgaaacgcgcgagacgaaagggcctcgtgatacgcct
atttttataggttaatgtcatgataataatggtttcttagacgtcaggtggcacttttcg
gggaaatgtgcgcggaacccctatttgtttatttttctaaatacattcaaatatgtatcc
gctcatgagacaataaccctgataaatgcttcaataatattgaaaaaggaagagtatgag
tattcaacatttccgtgtcgcccttattcccttttttgcggcattttgccttcctgtttt
tgctcacccagaaacgctggtgaaagtaaaagatgctgaagatcagttgggtgcacgagt
gggttacatcgaactggatctcaacagcggtaagatccttgagagttttcgccccgaaga
acgttttccaatgatgagcacttttaaagttctgctatgtggcgcggtattatcccgtat
tgacgccgggcaagagcaactcggtcgccgcatacactattctcagaatgacttggttga
gtactcaccagtcacagaaaagcatcttacggatggcatgacagtaagagaattatgcag
tgctgccataaccatgagtgataacactgcggccaacttacttctgacaacgatcggagg
accgaaggagctaaccgcttttttgcacaacatgggggatcatgtaactcgccttgatcg
ttgggaaccggagctgaatgaagccataccaaacgacgagcgtgacaccacgatgcctgt

agcaatggcaacaacgttgcgcaaactattaactggcgaactacttactctagcttcccg
gcaacaattaatagactggatggaggcggataaagttgcaggaccacttctgcgctcggc
ccttccggctggctggtttattgctgataaatctggagccggtgagcgtgggtctcgcgg
tatcattgcagcactggggccagatggtaagccctcccgtatcgtagttatctacacgac
ggggagtcaggcaactatggatgaacgaaatagacagatcgctgagataggtgcctcact
gattaagcattggtaactgtcagaccaagtttactcatatatactttagattgatttaaa
acttcatttttaatttaaaaggatctaggtgaagatcctttttgataatctcatgaccaa
aatcccttaacgtgagttttcgttccactgagcgtcagaccccgtagaaaagatcaaagg
atcttcttgagatcctttttttctgcgcgtaatctgctgcttgcaaacaaaaaaaccacc
gctaccagcggtggtttgtttgccggatcaagagctaccaactctttttccgaaggtaac
tggcttcagcagagcgcagataccaaatactgtccttctagtgtagccgtagttaggcca
ccacttcaagaactctgtagcaccgcctacatacctcgctctgctaatcctgttaccagt
ggctgctgccagtggcgataagtcgtgtcttaccgggttggactcaagacgatagttacc
ggataaggcgcagcggtcgggctgaacggggggttcgtgcacacagcccagcttggagcg
aacgacctacaccgaactgagatacctacagcgtgagcattgagaaagcgccacgcttcc
cgaagggagaaaggcggacaggtatccggtaagcggcagggtcggaacaggagagcgcac
gagggagcttccagggggaaacgcctggtatctttatagtcctgtcgggtttcgccacct
ctgacttgagcgtcgatttttgtgatgctcgtcaggggggcggagcctatggaaaaacgc
cagcaacgcggcctttttacggttcctggccttttgctggccttttgctcacatgttctt
tcctgcgttatcccctgattctgtggataaccgtattaccgcctttgagtgagctgatac
cgctcgccgcagccgaacgaccgagcgcagcgagtcagtgagcgaggaagcggaagagcg
cccaatacgcaaaccgcctctccccgcgcgttggccgattcattaatgcagctggcacga
caggtttcccgactggaaagcgggcagtgagcgcaacgcaattaatgtgagttagctcac
tcattaggcaccccaggctttacactttatgcttccggctcgtatgttgtgtggaattgt
gagcggataacaatttcacacaggaaacagctatgaccatgattacgaattcgagctcgg
tacccggggatcctctagagtcgacgctcgcgcgacttggtttgccattctttagcgcgc
gtcgcgtcacacagcttggccacaatgtggtttttgtcaaacgaagattctatgacgtgt
ttaaagtttaggtcgagtaaagcgcaaatctttttaaccctagaaagatagtctgcgta
aaattgacgcatgcattcttgaaatattgctctctctttctaaatagcgcgaatccgtcg
ctgtgcatttaggacatctcagtcgccgcttggagctcccgtgaggcgtgcttgtcaatg
cggtaagtgtcactgattttgaactataacgaccgcgtgagtcaaaatgacgcatgatta
tcttttacgtgacttttaagatttaactcatacgataattatattgttatttcatgttct
acttacgtgataacttattatatatatatttcttgttatagatatcgtgactaatatat
aataaaatgggtagttctttagacgatgagcatatcctctctgctcttctgcaaagcgat
gacgagcttgttggtgaggattctgacagtgaaatatcagatcacgtaagtgaagatgac
gtccagagcgatacagaagaagcgtttatagatgaggtacatgaagtgcagccaacgtca
agcggtagtgaaatattagacgaacaaaatgttattgaacaaccaggttcttcattggct
tctaacagaatcttgaccttgccacagaggactattagaggtaagaataaacattgttgg
tcaacttcaaagtccacgaggcgtagccgagtctctgcactgaacattgtcagatctcaa
agaggtccgacgcgtatgtgccgcaatatatatgacccacttttatgcttcaaactattt
tttactgatgagataatttcggaaattgtaaaatggacaaatgctgagatatcattgaaa
cgtcgggaatctatgacaggtgctacatttcgtgacacgaatgaagatgaaatctatgct
ttctttggtattctggtaatgacagcagtgagaaaagataaccacatgtccacagatgac
ctctttgatcgatctttgtcaatggtgtacgtctctgtaatgagtcgtgatcgttttgat
tttttgatacgatgtcttagaatggatgacaaaagtatacggcccacacttcgagaaaac
gatgtatttactcctgttagaaaaatatgggatctctttatccatcagtgcatacaaaat
tacactccaggggctcatttgaccatagatgaacagttacttggttttagaggacggtgt
ccgtttaggatgtatatcccaaacaagccaagtaagtatggaataaaaatcctcatgatg
tgtgacagtggtacgaagtatatgataaatggaatgccttatttgggaagaggaacacag
accaacggagtaccactcggtgaatactacgtgaaggagttatcaaagcctgtgcacggt
agttgtcgtaatattacgtgtgacaattggttcacctcaatccctttggcaaaaaactta
ctacaagaaccgtataagttcgagatcggccggcctagctcgaagaagttcctattccga
agttcctattctctagaaagtataggaacttccctaggataacttcgtataatgtatgct
atacgaagttatcctgcggcgcgccatgcggccgcgcgttaagatacattgatgagtttg
gacaaaccacaactagaatgcagtgaaaaaaatgctttatttgtgaaatttgtgatgcta
ttgctttatttgtaaccattataagctgcaataaacaagttaacaacaacaattgcattc
attttatgtttcaggttcagggggaggtgtgggaggttttttaaagcaagtaaaacctct
acaaatgtggtatggctgattatgatctgagtccggatattacgggtcctccaccttccg
ctttttcttgggtcgagatctcaggaacaggtggtggcggccctcggtgcgctcgtactg
ctccacgatggtgtagtcctcgttgtgggaggtgatgtccagcttggcgtccacgtagta
gtagccgggcagctgcacgggcttcttggccatgtagatggacttgaactccaccaggta

	gtggccgccgtccttcagcttcagggccttgtgggtctcgcccttcagcacgccgtcgcg<br>ggggtacaggcgctcggtggaggcctcccagcccatggtcttcttctgcatcacggggcc<br>gtcggaggggaagttcacgccgatgaacttcaccttgtagatgaagcagccgtcctgcag<br>ggaggagtcctgggtcacggtcgccacgccgccgtcctcgaagttcatcacgcgctccca<br>cttgaagccctcggggaaggacagcttcttgtagtcggggatgtcggcggggtgcttcac<br>gtacaccttggagccgtactggaactgggggacaggatgtcccaggcgaagggcagggg<br>gccgcccttggtcaccttcagcttcacggtgttgtggccctcgtagggcggccctcgcc<br>ctcgccctcgatctcgaactcgtggccgttcacggtgccctccatgcgcaccttgaagcg<br>catgaactcggtgatgacgttctcggaggaggccatggtggcgaccggtttgcgcttctt<br>cttgggtggggtgggatccaccagagacaggttgcggcggcggttggatggcgtgggcgc<br>gttggcgttgttggaccggctcatgttgtgtcgctgtaacagatgctgttcaactgtgtt<br>taccagatcgttgcgggctgtatttataggcgcgataagcgggacggcgcctcgtgtcc<br>ggtcacgcgcatgagataacgcgcggctgatatggaggcgcgtcctgttccgataaggag<br>ttgcgtccggctgcggttagcaacacaggaagctggcgtcctgtcacgataagacaacac<br>tcgtccggtccgataatgtgattcgtacgtgacaggacgcgacccgataaggccggccta<br>cgtgactgccgacacgtactttttttgcactgcaaaaaggttcaatgtgtggtagtgtatt<br>tggagcgtatacaacggtgtagactatttatgtaaaatagtctacgaaacgtagagtttg<br>tactatgtatgggcccgcgtgcaaaagcgtgtttttttgcagtgcaaaaaagttggtggt<br>ggggaggccaccgagtatgttaattaatatt
OX4580	ttgggctccccgggcgcgtactccacctcacccatctggtccatcatgatgaacgggtcg<br>aggtggcggtagttgatcccggcgaacgcgcggcgcaccgggaagccctcgccctcgaaa<br>ccgctgggcgcggtggtcacggtgagcacgggacgtgcgacggcgtcggcgggtgcggat<br>acgcggggcagcgtcagcgggttctcgacggtcacggcgggcatgtcgacggtatcgata<br>agcttgggccccccctcgaggttcccacaatggttaattcgagctccagcttttgttccc<br>tttagtgagggttaattgcgcgcttggcgtaatcatggtcatagctgtttcctgtgtgaa<br>attgttatccgctcacaattccacacaacatacgagccggaagcataaagtgtaaagcct<br>ggggtgcctaatgagtgagctaactcacattaattgcgttgcgctcactgcccgctttcc<br>agtcgggaaacctgtcgtgccagctgcattaatgaatcggccaacgcgcggggagaggcg<br>gtttgcgtattgggcgctcttccgcttcctcgctcactgactcgctgcgctcggtcgttc<br>ggctgcggcgagcggtatcagctcactcaaaggcggtaatacggttatccacagaatcag<br>gggataacgcaggaaagaacatgtgagcaaaaggccagcaaaaggccaggaaccgtaaaa<br>aggccgcgttgctggcgtttttccataggctccgcccccctgacgagcatcacaaaaatc<br>gacgctcaagtcagaggtggcgaaacccgacaggactataaagataccaggcgtttcccc<br>ctggaagctccctcgtgcgctctcctgttccgaccctgccgcttaccggatacctgtccg<br>cctttctcccttcgggaagcgtggcgctttctcatagctcacgctgtaggtatctcagtt<br>cggtgtaggtcgttcgctccaagctgggctgtgtgcacgaaccccccgttcagcccgacc<br>gctgcgccttatccggtaactatcgtcttgagtccaacccggtaagacacgacttatcgc<br>cactggcagcagccactggtaacaggattagcagagcgaggtatgtaggcggtgctacag<br>agttcttgaagtggtggcctaactacggctacactagaaggacagtatttggtatctgcg<br>ctctgctgaagccagttaccttcggaaaaagagttggtagctcttgatccggcaaacaaa<br>ccaccgctggtagcggtggtttttttgtttgcaagcagcagattacgcgcagaaaaaaag<br>gatctcaagaagatcctttgatcttttctacggggtctgacgctcagtggaacgaaaact<br>cacgttaagggattttggtcatgagattatcaaaaaggatcttcacctagatccttttaa<br>attaaaaatgaagttttaaatcaatctaaagtatatatgagtaaacttggtctgacagtt<br>accaatgcttaatcagtgaggcacctatctcagcgatctgtctatttcgttcatccatag<br>ttgcctgactccccgtcgtgtagataactacgatacgggagggcttaccatctggcccca<br>gtgctgcaatgataccgcgagacccacgctcaccggctccagatttatcagcaataaacc<br>agccagccggaagggccgagcgcagaagtggtcctgcaactttatccgcctccatccagt<br>ctattaattgttgccgggaagctagagtaagtagttcgccagttaatagtttgcgcaacg<br>ttgttgccattgctacaggcatcgtggtgtcacgctcgtcgtttggtatggcttcattca<br>gctccggttcccaacgatcaaggcgagttacatgatcccccatgttgtgcaaaaaagcgg<br>ttagctccttcggtcctccgatcgttgtcagaagtaagttggccgcagtgttatcactca<br>tggttatggcagcactgcataattctcttactgtcatgccatccgtaagatgcttttctg<br>tgactggtgagtactcaaccaagtcattctgagaatagtgtatgcggcgaccgagttgct<br>cttgcccggcgtcaatacgggataataccgcgccacatagcagaactttaaaagtgctca<br>tcattggaaaacgttcttcggggcgaaaactctcaaggatcttaccgctgttgagatcca<br>gttcgatgtaacccactcgtgcacccaactgatcttcagcatcttttactttcaccagcg<br>tttctgggtgagcaaaaacaggaaggcaaaatgccgcaaaaaagggaataagggcgacac<br>ggaaatgttgaatactcatactcttcctttttcaatattattgaagcatttatcagggtt<br>attgtctcatgagcggatacatatttgaatgtatttagaaaaataaacaaataggggttc

	cgcgcacatttccccgaaaagtgccacctaaattgtaagcgttaatattttgttaaaatt<br>cgcgttaaatttttgttaaatcagctcattttttaaccaataggccgaaatcggcaaaat<br>cccttataaatcaaaagaatagaccgagatagggttgagtgttgttccagtttggaacaa<br>gagtccactattaaagaacgtggactccaacgtcaaagggcgaaaaaccgtctatcaggg<br>cgatggcccactacgtgaaccatcaccctaatcaagttttttggggtcgaggtgccgtaa<br>agcactaaatcggaaccctaaagggagcccccgatttagagcttgacggggaaagccggc<br>gaacgtggcgagaaaggaagggaagaaagcgaaaggagcgggcgctagggcgctggcaag<br>tgtagcggtcacgctgcgcgtaaccaccacacccgccgcgcttaatgcgccgctacaggg<br>cgcgtcccattcgccattcaggctgcgcaactgttgggaagggctagctcgaagaagttc<br>ctattccgaagttcctattctctagaaagtataggaacttccctaggataacttcgtata<br>atgtatgctatacgaagttatcctgcggcgcgcctcgcgttaagatacattgatgagttt<br>ggacaaaccacaactagaatgcagtgaaaaaaatgctttatttgtgaaatttgtgatgct<br>attgctttatttgtaaccattataagctgcaataaacaagttaacaacaacaattgcatt<br>cattttatgtttcaggttcagggggaggtgtgggaggttttttaaagcaagtaaaacctc<br>tacaaatgtggtatggctgattatgatcagttatctagatccggtggatcttacgggtcc<br>tccaccttccgctttttcttgggtcgagatctgagtccggagggcaaggcggagccggag<br>gcgatggcgtgctcggtcaggtgccacttctggttcttggcgtcgctgcggtcctcgcgg<br>gtcagcttgtgctggatgaagtgccagtcgggcatcttgcggggcacggacttggccttg<br>tacacggtgtcgaactggcagcgcaagcggccaccgtccttcagcagcaggtacatgctc<br>acgtcgcccttcaagatgccctgcttgggcacggggatgatcttctcgcaggagggctcc<br>cagttgtcggtcatcttcttcatcacggggccgtcggcggggaagttcacgccgtagaac<br>ttggactcgtggtacatgcagttctcctccacgctcacggtgatgtcggcgttgcagatg<br>cacacggcgccgtcctcgaacaggaaggagcggtcccaggtgtagccggcggggcaggag<br>ttcttgaagtagtcgacgatgtcctggggggtactcggtgaacacgcggttgccgtacatg<br>aaggcggcggacaagatgtcctcggcgaagggcaagggggccgccctccaccacgcacagg<br>ttgatggcctgcttgcccttgaaggggtagccgatgccctcgccggtgatcacgaacttg<br>tggccgtccacgcagccctccatgcggtacttcatggtcatctccttggtcaggccgtgc<br>ttggactgggccatggtggcgaccggtttgcgcttcttcttgggtggggtgggatctccc<br>atggtggcctgaatctcaacttgcacctgaaggtagtgcagcaaggatgagcaaaaggga<br>agaacccagaaaagaacgggaaaacttaccccaattagaattgcttgtcgccgccagtgt<br>caacttgcaactgaaacaatatccaacatgaacgtcaatttatactgccctaatggcgaa<br>cacgataacaatatttcttttattatgccctctaaaccaacgcggttatcgtttattta<br>ttcaaattagatatagaacatccgccgacatacaatgttaatgcaaaaacgcgtttggtg<br>agcggatacgaaaacagtcggccgataaacattaatctgaggtcggtaacaccgtccttg<br>aacggaacacgaggagcgtacgtgatcagctgcattcgcgcgccgcgcctttatcgagat<br>ttatttgcatacaacaagtacactgcgccgttgggatttgtggtaacgcgcacacatgca<br>gagctgcaagtgtggcacattttgtctgtgcgcaaacctttgaagccaaaagtacgagg<br>tccgttacgggcatgctagcgcacacggacaatggacccgacaaattctacgccaaggat<br>ttaatgataatgtcgggcaacgtatccgttcattttatcaataacctacaaaaatgtcgc<br>gcgcatcacaaagacatcgatatatttaaacatttatgtcccgaactgcaaatcgataat<br>agtgttgtgcaacctcgagcgtccgtttgatttaacgtatagcttgcaaatgaattattt<br>aattatcaatcatgttttacgcgtagaattctacccgtaaagcgagtttagttatgagcc<br>atgtgcaaaacatgacatcagcttttatttttataacaaatgacatcatttcttgattgt<br>gttttacacgtagaattctactcgtaaagcgagttcagttttgaaaaacaaatgacatca<br>tctttttgattgtgctttacaagtagaattctacccgtaaatcaagttcggttttgaaaa<br>acaaatgagtcatattgtatgatatcatattgcaaaacaaatgactcatcaatcgatcgt<br>gcgttacacgtagaattctactcgtaaagcgagtttatgagccgtgtgcaaaacatgaca<br>tcatctcgatttgaaaaacaaatgacatcatccactgatcgtgcgttacaagtagaattc<br>tactcgtaaagccagttcggttatgagccgtgtgcaaaacatgacatcagcttatgactc<br>atacttgattgtgttttacgcgtagaattctactcgtaaagccagttcaattttaaaaac<br>aaatgacatcatccaaattaataaatgacaagcaatggcggccgcacgcgatcggtgcgg<br>gcctcttcgctattacgccagctggcgaaagggggatgtgctgcaaggcgattaagttgg<br>gtaacgccagggttttcccagtcacgacgttgtaaaacgacggccagtgagcgcgcgtaa<br>tacgactcactatagggcgaattgggtaccgggccccccctcgaggtcgacgatgtaggt<br>cacggtctcgaagccgcggtgcgggtgccagggcgtgccc
OX4714	ttgggctccccgggcgcgtactccacctcacccatctggtccatcatgatgaacgggtcg<br>aggtggcggtagttgatcccggcgaacgcgcggcgcaccgggaagccctcgccctcgaaa<br>ccgctgggcgcggtggtcacggtgagcacgggacgtgcgacggcgtcggcgggtgcggat<br>acgcggggcagcgtcagcgggttctcgacggtcacggcgggcatgtcgacggtatcgata<br>agcttgggccccccctcgaggttcccacaatggttaattcgagctccagcttttgttccc

tttagtgagggttaattgcgcgcttggcgtaatcatggtcatagctgtttcctgtgtgaa
attgttatccgctcacaattccacacaacatacgagccggaagcataaagtgtaaagcct
ggggtgcctaatgagtgagctaactcacattaattgcgttgcgctcactgcccgctttcc
agtcgggaaacctgtcgtgccagctgcattaatgaatcggccaacgcgcggggagaggcg
gtttgcgtattgggcgctcttccgcttcctcgctcactgactcgctgcgctcggtcgttc
ggctgcggcgagcggtatcagctcactcaaaggcggtaatacggttatccacagaatcag
gggataacgcaggaaagaacatgtgagcaaaaggccagcaaaaggccaggaaccgtaaaa
aggccgcgttgctggcgtttttccataggctccgcccccctgacgagcatcacaaaaatc
gacgctcaagtcagaggtggcgaaacccgacaggactataaagataccaggcgtttcccc
ctggaagctccctcgtgcgctctcctgttccgaccctgccgcttaccggatacctgtccg
cctttctcccttcgggaagcgtggcgctttctcatagctcacgctgtaggtatctcagtt
cggtgtaggtcgttcgctccaagctgggctgtgtgcacgaaccccccgttcagcccgacc
gctgcgccttatccggtaactatcgtcttgagtccaacccggtaagacacgacttatcgc
cactggcagcagccactggtaacaggattagcagagcgaggtatgtaggcggtgctacag
agttcttgaagtggtggcctaactacggctacactagaaggacagtatttggtatctgcg
ctctgctgaagccagttaccttcggaaaaagagttggtagctcttgatccggcaaacaaa
ccaccgctggtagcggtggtttttttgtttgcaagcagcagattacgcgcagaaaaaaag
gatctcaagaagatcctttgatcttttctacggggtctgacgctcagtggaacgaaaact
cacgttaagggattttggtcatgagattatcaaaaaggatcttcacctagatccttttaa
attaaaaatgaagttttaaatcaatctaaagtatatatgagtaaacttggtctgacagtt
accaatgcttaatcagtgaggcacctatctcagcgatctgtctatttcgttcatccatag
ttgcctgactccccgtcgtgtagataactacgatacgggagggcttaccatctggcccca
gtgctgcaatgataccgcgagacccacgctcaccggctccagatttatcagcaataaacc
agccagccggaagggccgagcgcagaagtggtcctgcaactttatccgcctccatccagt
ctattaattgttgccgggaagctagagtaagtagttcgccagttaatagtttgcgcaacg
ttgttgccattgctacaggcatcgtggtgtcacgctcgtcgtttggtatggcttcattca
gctccggttcccaacgatcaaggcgagttacatgatcccccatgttgtgcaaaaaagcgg
ttagctccttcggtcctccgatcgttgtcagaagtaagttggccgcagtgttatcactca
tggttatggcagcactgcataattctcttactgtcatgccatccgtaagatgcttttctg
tgactggtgagtactcaaccaagtcattctgagaatagtgtatgcggcgaccgagttgct
cttgcccggcgtcaatacgggataataccgcgccacatagcagaactttaaaagtgctca
tcattggaaaacgttcttcggggcgaaaactctcaaggatcttaccgctgttgagatcca
gttcgatgtaacccactcgtgcacccaactgatcttcagcatcttttactttcaccagcg
tttctgggtgagcaaaaacaggaaggcaaaatgccgcaaaaaagggaataagggcgacac
ggaaatgttgaatactcatactcttcctttttcaatattattgaagcatttatcagggtt
attgtctcatgagcggatacatatttgaatgtatttagaaaaataaacaaataggggttc
cgcgcacatttccccgaaaagtgccacctaaattgtaagcgttaatattttgttaaaatt
cgcgttaaatttttgttaaatcagctcattttttaaccaataggccgaaatcggcaaaat
cccttataaatcaaaagaatagaccgagatagggttgagtgttgttccagtttggaacaa
gagtccactattaaagaacgtggactccaacgtcaaagggcgaaaaaccgtctatcaggg
cgatggcccactacgtgaaccatcaccctaatcaagttttttggggtcgaggtgccgtaa
agcactaaatcggaaccctaaagggagcccccgatttagagcttgacggggaaagccggc
gaacgtggcgagaaaggaagggaagaaagcgaaaggagcgggcgctagggcgctggcaag
tgtagcggtcacgctgcgcgtaaccaccacacccgccgcgcttaatgcgccgctacaggg
cgcgtcccattcgccattcaggctgcgcaactgttgggaagggctagctcgaagaagttc
ctattccgaagttcctattctctagaaagtataggaacttccctaggataacttcgtata
atgtatgctatacgaagttatcctgcggcgcgcccgcgttaagatacattgatgagtttg
gacaaaccacaactagaatgcagtgaaaaaaatgctttatttgtgaaatttgtgatgcta
ttgctttatttgtaaccattataagctgcaataaacaagttaacaacaacaattgcattc
attttatgtttcaggttcagggggaggtgtgggaggttttttaaagcaagtaaaacctct
acaaatgtggtatggctgattatgatcagttatctagatccggtggatcttacgggtcct
ccaccttccgctttttcttgggtcgagatctcaggaacaggtggtggcggccctcggtgc
gctcgtactgctccacgatggtgtagtcctcgttgtgggaggtgatgtccagcttggcgt
ccacgtagtagtagccgggcagctgcacgggcttcttggccatgtagatggacttgaact
ccaccaggtagtggccgccgtccttcagcttcagggccttgtgggtctcgcccttcagca
cgccgtcgcgggggtacaggcgctcggtggaggcctcccagcccatggtcttcttctgca
tcacggggccgtcggaggggaagttcacgccgatgaacttcaccttgtagatgaagcagc
cgtcctgcagggaggagtcctgggtcacggtcgccacgccgccgtcctcgaagttcatca
cgcgctcccacttgaagccctcggggaaggacagcttcttgtagtcggggatgtcggcgg
ggtgcttcacgtacaccttggagccgtactggaactggggggacaggatgtcccaggcga
agggcagggggccgcccttggtcaccttcagcttcacggtgttgtggccctcgtaggggc

	ggccctcgccctcgccctcgatctcgaactcgtggccgttcacggtgccctccatgcgca<br>ccttgaagcgcatgaactcggtgatgacgttctcggaggaggccatggtggcgaccggtt<br>tgcgcttcttcttgggtggggtgggatctcccatggtggcctattcatggtggcgaccgg<br>tggatcgtaccgtcgactctagcggtaccccgattgtttagcttgttcagctgcgcttgt<br>ttatttgcttagctttcgcttagcgacgtgttcactttgcttgtttgaattgaattgtcg<br>ctccgtagacgaagcgcctctatttatactccggcggtcgagggttcgaaatcgataagc<br>ttggatcctaattgaattagctctaattgaattagtctctaattgaattagatccccaaa<br>tctggccggccgcacgcgatcggtgcgggcctcttcgctattacgccagctggcgaaagg<br>gggatgtgctgcaaggcgattaagttgggtaacgccagggttttcccagtcacgacgttg<br>taaaacgacggccagtgagcgcgcgtaatacgactcactatagggcgaattgggtaccgg<br>gccccccctcgaggtcgacgatgtaggtcacggtctcgaagccgcggtgcgggtgccagg<br>gcgtgccc

Table B. Oligonucleotides for the adaptor-based amplification of flanking genomic DNA.

Name	Type (specificity)	Sequence 5'-3'
Adaptor_long	Adaptor	GTGTAGCGTGAAGACGACAGAAGGGC GTGGTGCGGAGGGCGGTG
MspI(TaqI)_ short	Adaptor	CGCACCGCCCTCCG
DpnII_short	Adaptor	GATCCACCGCCCTCCG
PRIMER	Primer (Adaptor)	GTGTAGCGTGAAGACGACAGAA
PB1	Primer (5' *piggyBac* end)	GGCGACTGAGATGTCCTAAATGCAC
PB2	Primer (5' *piggyBac* end)	CAGTGACACTTACCGCATTGACAAG
PB3	Primer (3' *piggyBac* end)	CAGACCGATAAAACACATGCGTCA
PB4	Primer (3' *piggyBac* end)	GTGCCAAAGTTGTTTCTGACTGACTA
MID	Primer (Adaptor)	GACGACAGAAAGGGCGTGGTG
pJETFP2	Primer (pJET vector)	ATCAACTGCTTTAACACTTGTGC
pJETRP2	Primer (pJET vector)	AAAGAAGAACATCGATTTTCCATG

Table C. Oligonucleotides for identification of recombination and excision events. Expected amplicon sizes and the full 5'-3' sequences are included.

Amplicon [size]	Forward Primer name and sequence 5'-3'	Reverse Primers name and sequence 5'-3'
Aedes aegypti ΦC31-RMCE		
5' *attP*-DsRed junction [579 bp]	P1 (*attP*-Diag1): GTAACCTTTGAGTTCTCTCAGTTGG	P2 (DsRed-5'): CTCGATCTCGAACTCGTGGC
3' DsRed-*attP* junction [1179 bp]	P3 (DsRed-3'): CGGGGTACCGCTAGAGTCG	P1 (*attP*-Diag1): GTAACCTTTGAGTTCTCTCAGTTGG
5' *attR*-AmCyan junction [579 bp]	P4 (*attR*-Diag1): TGGGGTAACCTTTGGGCTCC	P5 (AmCyan-5'): TGCAGAGCTGCAAGTGTGGC
3' AmCyan-*attR* junction [1179 bp]	P6 (Amcyan-3'): CTTGAAGGGCGACGTGACCGCC	P4 (*attR*-Diag1): TGGGGTAACCTTTGGGCTCC
Aedes aegypti iRMCE		
loxP/FRT-DsRed2 [500 bp]	FRTAscF: TCGAAGAAGTTCCTATTCCGAAGTTCC	RedSeqF: AAGGGCGAGACCCACAAGG
attL [491 bp]	Diag-recomb: CGACTCACTATAGGGCGAATTGG	Diag3pb-xho: TCATCTGATGTACCAGGCACTTC
attR [394 bp]	AttPPstF: GTTCTGTGATGACCTGCAGCCCG	AttBXhoR: GGTGTGCTCGAGAAGCTTATCGATACCGTCGACATG
OX4476C 5' genomic-*pBac* junction [1084 bp]	AttPFlF1: TGGGATTCGATTCGAACTCATG	PB1: GGCGACTGAGATGTCCTAAATGCAC
OX4476F 5' genomic-*pBac* junction [498 bp]	AttPFlF3: GTTGATGGCGGTGCGACAATATAAC	PB1: GGCGACTGAGATGTCCTAAATGCAC
pBac-DsRed2: across excision junction; if excision: [845 bp] if not: [5556 bp]	Seq-transp-3: CCTCATGATGTGTGACAGTGGTAC	RedseqF: AAGGGCGAGACCCACAAGG
Plutella xylostella iRMCE		
attL [425 bp]	Int2: TCTCCCTTGCTACTGACATTATGGCTG	Int3: CTGCAAGGCGATTAAGTTGGGTAAC
attR [380 bp]	Int 1: ACACGCTAGACCAAATGTGTTCTGTG	Int 4: CAGTTCGGTTATGAGCCGTGTGC

AmpR [250 bp]	AmpRF: TCTCAGCGATCTGTCTATTTCGTTCATC	AmpRR: ATGCCTGTAGCAATGGCAACAACG
Opie2 [202 bp]	CTACGTGACTGCCGACACGTAC	CATACTCGGTGGCCTCCCCAC
ie1 [555 bp]	CTATTATCGATTTGCAGTTCGG	CCCTAATGGCGAACACGATAACAATA
attP [345 bp]	INT1: ACACGCTAGACCAAATGTGTTCTGTG	INT2: TCTCCCTTGCTACTGACATTATGGCTG
